# Spin‐Polarized Surface Capacitance Effects Enable Fe_3_O_4_ Anode Superior Wide Operation‐Temperature Sodium Storage

**DOI:** 10.1002/advs.202306992

**Published:** 2023-12-07

**Authors:** Zhenwei Li, Meisheng Han, Peilun Yu, Jie Yu

**Affiliations:** ^1^ Guangdong Provincial Key Laboratory of Semiconductor Optoelectronic Materials and Intelligent Photonic Systems Shenzhen Engineering Lab for Supercapacitor Materials School of Material Science and Engineering Harbin Institute of Technology, Shenzhen University Town Shenzhen 518055 China; ^2^ Songshan Lake Materials Laboratory Dongguan Guangdong 523808 China; ^3^ Department of Mechanical and Energy Engineering Southern University of Science and Technology Shenzhen 518055 China

**Keywords:** Fe_3_O_4_ anode, sodium‐ion batteries, spin‐polarized surface capacitance effect, wide operation‐temperature

## Abstract

Fe_3_O_4_ is widely investigated as an anode for ambient sodium‐ion batteries (SIBs), but its electrochemical properties in the wide operation‐temperature range have rarely been studied. Herein, the Fe_3_O_4_ nanoparticles, which are well encapsulated by carbon nanolayers, are uniformly dispersed on the graphene basal plane (named Fe_3_O_4_/C@G) to be used as the anode for SIBs. The existence of graphene can reduce the size of Fe_3_O_4_/C nanoparticles from 150 to 80 nm and greatly boost charge transport capability of electrode, resulting in an obvious size decrease of superparamagnetic Fe nanoparticles generated from the conversion reaction from 5 to 2 nm. Importantly, the ultra‐small superparamagnetic Fe nanoparticles (≈2 nm) can induce a strong spin‐polarized surface capacitance effect at operating temperatures ranging from −40 to 60 °C, thus achieving highly efficient Na‐ion transport and storage in a wide operation‐temperature range. Consequently, the Fe_3_O_4_/C@G anode shows high capacity, excellent fast‐charging capability, and cycling stability ranging from −40 to 60 °C in half/full cells. This work demonstrates the viability of Fe_3_O_4_ as anode for wide operation‐temperature SIBs and reveals that spin‐polarized surface capacitance effects can promote Na‐ion storage over a wide operation temperature range.

## Graphical abstract

Fe_3_O_4_ is widely investigated as an anode for ambient SIBs, but its sodium storage performance in wide operation‐temperature range has rarely been studied. This work reveals that Fe nanoparticles generated from Fe_3_O_4_ in conversion reaction induce strong spin‐polarized surface capacitance effect to boost sodium storage over a wide operating temperature of −40–60 °C.

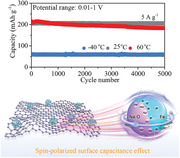

## 1 Introduction

Lithium‐ion batteries (LIBs) have become an indispensable part of human life since their successful commercialization in the 1990s.^[^ ^1^ ^]^ Currently, the rapid development of electric vehicles has greatly increased the demand for LIBs, which has raised serious concerns about the limited lithium reserves.^[^ ^2^ ^]^ Sodium‐ion batteries (SIBs) working with a similar mechanism to that of LIBs are considered a promising alternative/supplement due to the abundant resources, wide distribution in the earth, and low cost of sodium.^[^ ^3^ ^]^ However, the ionic radius of Na^+^ (1.02 Å) is much larger than that of Li^+^ (0.76 Å). It is undeniable that the larger radius of Na^+^ leads to sluggish Na‐ion diffusion dynamics and severe pulverization of electrode material, thus resulting in poor rate performance and unsatisfactory cycling stability.^[^ ^4^ ^]^ Many efforts have been made to develop suitable anode materials for SIBs over the past ten years, such as hard carbon, graphene, Fe_3_O_4_, TiO_2_, MoS_2_, FeS_2_, etc.^[^ ^5^, ^6^, ^7^, ^8^, ^9^, ^10^, ^11^, ^12^ ^]^ among which, Fe_3_O_4_ has been the subject of intensive research because of its high sodium storage capacity, unique conversion mechanism, and abundant reserves, as well as environmental friendliness. Its electrochemical property is poor, however, due to the huge volume change upon cycling and poor intrinsic conductivity.

Up to date, many efforts have been made to address these issues. One strategy is to synthesize nanostructured Fe_3_O_4_.^[^ ^13^, ^14^, ^15^ ^]^ The nanoscale particle size not only improves the accommodation of the strain but also significantly shortens the length of the diffusion path of Na^+^. This is critical for Na‐ion transport and storage, especially considering the large ionic radius of Na^+^. However, the decrease in particle size leads to a significant increase in interfacial charge transfer resistance and severe agglomeration of Fe_3_O_4_ nanoparticles, which results in a degradation of cell performance. Another strategy is to construct Fe_3_O_4_–carbon composites.^[^ ^16^, ^17^, ^18^ ^]^ This strategy not only improves electrode conductivity, but also suppresses volume expansion/contraction and buffers the agglomeration of Fe_3_O_4_. However, the conversion reaction causes a dramatic and irreversible structural change in Fe_3_O_4_, which may make the original structure design incapable of matching the electrode system after the conversion reaction, thus degrading cell performance.

In addition, the electrochemical performance of Fe_3_O_4_‐based electrodes for SIBs at room temperature has been widely studied. Its electrochemical performance at low and high temperatures, however, has received little attention, which is extremely important during practical applications. Especially at low temperatures, the Na‐ion diffusion kinetics in the active material are greatly suppressed, resulting in significant degradation of the overall electrochemical performance of SIBs and posing a great challenge for the use of SIBs in cold climates.^[^ ^19^ ^]^ As a typical conversion‐type anode, Fe_3_O_4_ generates a large number of superparamagnetic Fe nanoparticles during the conversion reaction.^[^ ^20^ ^]^ According to recent reports, the superparamagnetic 3d transition metal nanoparticles in the electrode can induce strong surface capacitance effect to improve ion transport and storage.^[^ ^21^, ^22^ ^]^ Specifically, the d‐orbit electrons of these transition metals are not full, once the ultrasmall 3d transition metal nanoparticles are formed during the electrochemical reaction, numerous spin‐polarized electrons are injected into their d orbits under the electric field.^[^ ^22^ ^]^ Meanwhile, Li^+^/Na^+^ can be stored in Li/Na‐ion conductor matrix (such as Li_2_O and Na_2_O). The spin‐polarized electrons and Li^+^/Na^+^ can attract each other and accumulate in their interface, resulting in the construction of the space charge zones and thereby inducing the spin‐polarized surface capacitance to store extra Li^+^/Na^+^.^[^ ^22^ ^]^ In addition, Li et al.^[^ ^23^ ^]^ confirmed that the spin‐polarized surface capacitance of the transition metal nanoparticles depends largely on the size of the nanoparticles. However, relevant studies are currently limited to LIBs/SIBs operating at room temperature. The effect of superparamagnetic transition metal nanoparticles on the transport and storage of Na^+^ at ultra‐low and high temperatures remains unknown. Note that the superparamagnetism of transition metal nanoparticles can be significantly enhanced at low temperatures compared to room temperature.^[^ ^24^ ^]^ It is reasonable to believe that the superparamagnetic Fe nanoparticles produced by the conversion reaction can induce a strong surface capacitance effect to enhance the diffusion kinetics of Na^+^ at low temperatures. However, to our knowledge, no relevant studies have been reported to date. Therefore, exploring surface capacitance effects on sodium ion transport and storage at ultra‐low and high temperatures, and using this to develop a robust Fe_3_O_4_‐carbon structure that can support highly efficient electron transport, withstand the huge shock from the conversion reaction, and perform excellent sodium storage in all‐weather environments are of great significance for the development of Fe_3_O_4_ electrodes.

Here, the Fe_3_O_4_ nanoparticles, which are well encapsulated by carbon nanolayers, are uniformly dispersed on the graphene basal plane (named Fe_3_O_4_/C@G) to be used as the anode for SIBs. The prepared Fe_3_O_4_/C@G possesses the following advantages: 1) Superparamagnetic Fe nanoparticles generated from Fe_3_O_4_ in conversion reactions induce strong surface‐capacitance effects on the Fe surface due to the injection of abundant spin‐polarized electrons, which boosts Na‐ion transport and storage, especially at low temperatures. 2) The introduction of graphene decreases the size of the superparamagnetic Fe nanoparticles, enhancing the spin‐polarized surface capacitance effect. 3) Graphene carries the Fe_3_O_4_/C and superparamagnetic Fe nanoparticles, avoiding the devastating damage to electrode caused by conversion reactions and providing a highly efficient electron transfer platform. 4) The carbon nanolayers alleviate the volume expansion of Fe_3_O_4_, enhance conductivity, and inhibit the agglomeration of Fe_3_O_4_ nanoparticles. Based on these features, the Fe_3_O_4_/C@G displays an ultrafast Na^+^ transport capability and long cycling life over a wide operation temperature range in half and full cells.

## 2 Results and Discussion

### 2.1 Materials Synthesis and Characterizations

**Figure 1:**
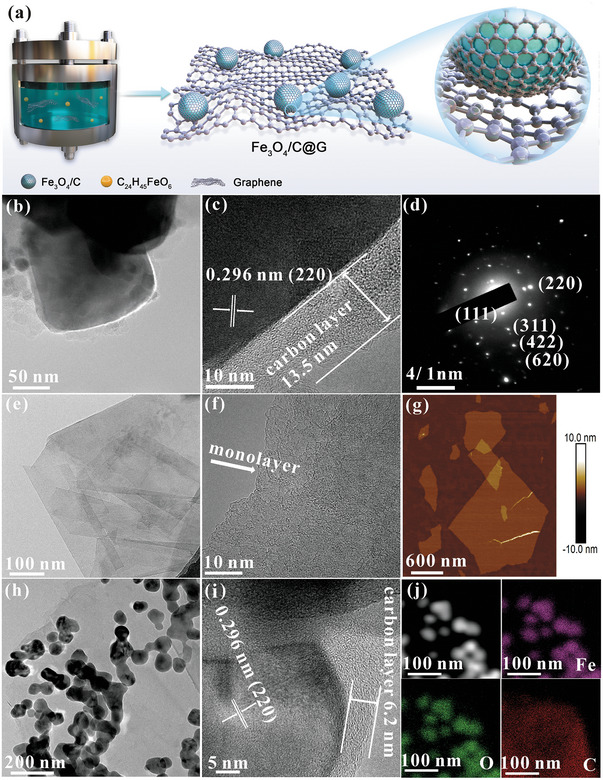
Structural characterization of samples. a) Schematic of the synthesis process of samples. b) TEM, c) HRTEM, d) SAED images of Fe_3_O_4_/C. e) TEM, f) HRTEM, and g) atomic force microscopy (AFM) images of the graphene. h) TEM, i) HRTEM, j) HAADF, and EDS images of the Fe_3_O_4_/C@G‐II.

**Figure** **1**a shows the schematic diagram of the synthetic process of samples with a one‐step pressure‐induced vapor synthetic method. Graphene is first uniformly dispersed into the iron(III) 2‐ethylhexanoate liquid by stirring for 0.5 h, which then is transferred and sealed within the reaction vessel at predetermined graphene/iron(III) 2‐ethylhexanoate ratios (1.5 mL/0 g, Fe_3_O_4_/C; 1.5 mL/0.01 g, Fe_3_O_4_/C@G‐I; and 3 mL/0.01 g, Fe_3_O_4_/C@G‐II). As the reaction temperature surpasses the boiling point of iron(III) 2‐ethylhexanoate (228 °C), the iron(III) 2‐ethylhexanoate rapidly vaporizes, leading to an increase in pressure within the sealed container. When the temperature is further increased to 500 °C and kept for 30 min, iron(III) 2‐ethylhexanoate gradually decomposes under high‐temperature and elevated gas‐phase pressure conditions. Possible decomposition products of CH_3_(CH_2_)_3_CH(C_2_H_5_)COO^−^, such as ·CH_3_, ·CH_2_, and other carbon‐based groups, transform into carbon nanolayers, while Fe^3+^ and some of the O in CH_3_(CH_2_)_3_CH(C_2_H_5_)COO^−^ converts into Fe_3_O_4_. Carbon nanolayer‐encapsulated Fe_3_O_4_ nanoparticles (Fe_3_O_4_/C) are synthesized in this process. After introducing graphene, Fe_3_O_4_/C nanoparticles are efficiently carried and dispersed on the graphene basal plane to obtain Fe_3_O_4_/C@G samples. Furthermore, it is worth noting that when the reaction temperature is ≤ 470 °C, the postreaction iron(III) 2‐ethylhexanoate remains in a liquid state without the formation of any solid phases. Elevating the reaction temperature to 475 °C results in the complete transformation of iron(III) 2‐ethylhexanoate from a liquid phase to a solid phase. X‐ray diffraction (XRD) pattern clearly reveals diffraction peaks characteristic of Fe_3_O_4_ (Figure S1, Supporting Information), indicating that the pyrolysis of iron(III) 2‐ethylhexanoate and the generation of Fe_3_O_4_ commence at approximately 475 °C. The morphology and internal structure of the samples are shown in Figure 1b–j and Figures S2–S5 (Supporting Information). As can be seen from the scanning electron microscope (SEM, Figure S2a–c, Supporting Information) and transmission electron microscopy (TEM, Figure 1b and Figure S3, Supporting Information) images, Fe_3_O_4_/C exhibits a blocky morphology with a size of over 150 nm. High‐resolution TEM (HRTEM) image (Figure 1c) shows a *d*‐spacing of 0.296 nm corresponding to the (220) reflection of Fe_3_O_4_, denoting the formation of Fe_3_O_4_. Meanwhile, the carbon nanolayers with a thickness of ≈13.5 nm well encapsulate the Fe_3_O_4_ nanoparticles. The selected area electron diffraction (SAED) pattern in Figure 1d displays clear diffraction spots and diffraction rings ([111], [220], [311], [422], and [620]), signaling the polycrystalline nature of Fe_3_O_4_. Figure 1e–g shows the features of graphene, which has a size of ≈1.5 µm and thickness of a single atomic layer. Figure 1h and Figures S2d–i, S4, and S5 (Supporting Information) exhibit the morphology of Fe_3_O_4_/C@G samples, as can be seen, numerous nanoparticles with a size of ≈80 nm are uniformly dispersed on the graphene basal plane. The obvious difference between Fe_3_O_4_/C@G‐I and Fe_3_O_4_/C@G‐II is that the surface of Fe_3_O_4_/C@G‐II is loaded with more Fe_3_O_4_ nanoparticles. This is because the reaction system used to synthesize Fe_3_O_4_/C@G‐II has more precursors of Fe_3_O_4_/C nanoparticles, i.e., the Iron(III) 2‐ethylhexanoate. HRTEM observation confirms the internal Fe_3_O_4_ and the external carbon nanolayers (Figure 1i). The thickness of the carbon nanolayers surrounding the Fe_3_O_4_ nanoparticles is about 6.2 nm. Compared to the pure Fe_3_O_4_/C sample (Figure 1b,c), a significant reduction in both the size of the Fe_3_O_4_/C and the thickness of the carbon nanolayer is observed after the introduction of graphene. The size decrease of Fe_3_O_4_ may be ascribed to the increase in nucleation rate and sites of Fe_3_O_4_ due to the presence of solid‐phase graphene. The thinner carbon nanolayer in Fe_3_O_4_/C@G may be attributed to the decrease of vapor pressure due to the presence of solid‐phase graphene, which would reduce the conversion of vapor‐phase carbon‐containing groups produced by pyrolysis of Iron(III) 2‐ethylhexanoate into solid carbon nanolayers under the sealed vessel. In addition to hosting Fe_3_O_4_/C nanoparticles, graphene supports efficient electron transport due to its ultra‐high electrical conductivity. The high‐angle annular dark‐field (HAADF) and the corresponding EDS elemental mapping images indicate that Fe_3_O_4_/C nanoparticles are homogeneously dispersed on the graphene basal plane, as shown in Figure 1j. As a result, the Fe_3_O_4_/C@G, with carbon nanolayer‐encapsulated Fe_3_O_4_ nanoparticles uniformly dispersed on graphene basal plane, is successfully fabricated.

**Figure 2:**
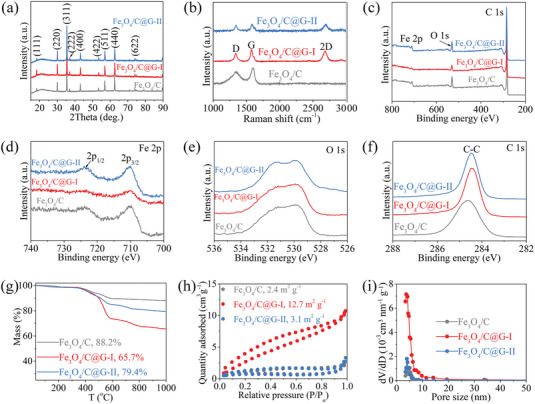
Structure analysis of samples. a) XRD patterns, b) Raman spectra, c) XPS spectra, d) Fe 2p spectra, e) O 1s spectra, f) C 1s spectra, g) TG curves, h) nitrogen adsorption/desorption isotherms, and i) pore size distribution of Fe_3_O_4_/C, Fe_3_O_4_/C@G‐I, and Fe_3_O_4_/C@G‐II.

The crystal structure of the prepared samples is analyzed by XRD, as shown in **Figure** **2**a. All samples show clear peaks at 18.3°, 30.1°, 35.4°, 37.1°, 43.2°, 53.5°, 57.0°, 62.5°, and 74.0°, corresponding to the (111), (220), (311), (222), (400), (422), (511), (440), and (622) planes of Fe_3_O_4_, respectively. This result indicates the formation of Fe_3_O_4_ in Fe_3_O_4_/C, Fe_3_O_4_/C@G‐I, and Fe_3_O_4_/C@G‐II. Furthermore, a broad peak corresponding to the amorphous carbon phase can be observed at ≈21° (partial enlarged detail in Figure S6, Supporting Information), indicating the amorphous nature of the carbon nanolayer encapsulating Fe_3_O_4_. This is consistent with the HRTEM observations. Figure 2b exhibits the Raman spectra of the prepared samples. Two typical carbon bands can be detected in all samples. The G band at ≈1596 cm^−1^ relates to the in‐plane stretching vibrations of the sp^2^ carbon atoms, and the D band at ≈1354 cm^−1^ is associated with lattice defects of carbon atoms.^[^ ^25^ ^]^ In addition to these two peaks, Fe_3_O_4_/C@G‐I and Fe_3_O_4_/C@G‐II also show a strong 2D band (≈2684 cm^−1^), further demonstrating the monolayer feature and no agglomeration occurs of graphene in these samples, in agreement with the TEM and XRD results above.

X‐ray photoelectron spectroscopy (XPS) is carried out to analyze the characteristics of elements and chemical states of the samples. The full survey XPS spectra (Figure 2c) show that all the expected elements (Fe, O, and C) are detected in Fe_3_O_4_/C, Fe_3_O_4_/C@G‐I, and Fe_3_O_4_/C@G‐II samples. The high‐resolution Fe 2p (Figure 2d; Figure S7a, Supporting Information) and O 1s (Figure 2e; Figure S7b, Supporting Information) spectra of all the prepared samples exhibit typical XPS peaks, such as 723.8 eV for Fe 2p_1/2_ and 710.1 eV for Fe 2p_3/2_; ≈531 eV for O 1s of Fe_3_O_4_,^[^ ^26^, ^27^ ^]^ further evidencing the formation of Fe_3_O_4_. In the C 1s spectra of samples (Figure 2f), the presence of graphene (C═C, sp^2^) in Fe_3_O_4_/C@G‐I and Fe_3_O_4_/C@G‐II induces a rightward shift of the C 1s peak compared to the amorphous carbon nanolayers (C─C, sp^3^) of Fe_3_O_4_/C.^[^ ^28^ ^]^ To assess the purity of the carbon nanolayers, we subjected the C1s spectrum of Fe_3_O_4_/C to peak deconvolution (Figure S8, Supporting Information). It can be observed that, in addition to the dominant C─C bonds, there are also some C─O bonds within Fe_3_O_4_/C, indicating the involvement of partial O from CH_3_(CH_2_)_3_CH(C_2_H_5_)COO^−^ in the construction of the carbon nanolayers. The presence of C─O bonds undoubtedly increases the defect concentration of the carbon nanolayers, which is further confirmed by the high *I*_D_/*I*_G_ ratio (0.84) in the Raman spectrum of the Fe_3_O_4_/C sample (Figure 2b). Figure 2g displays the thermogravimetric (TG) curves of these samples. Based on the residual mass after heating to 1000 °C in air (C→CO_2_), the Fe_3_O_4_ content in Fe_3_O_4_/C, Fe_3_O_4_/C@G‐I, and Fe_3_O_4_/C@G‐II can be measured to be 88.2, 65.7, and 79.4 wt.%, respectively. Figures 2h,i show the N_2_ adsorption–desorption profiles and pore size distribution of these samples, respectively. All samples show a mesoporous structure, where the specific surface area of Fe_3_O_4_/C@G‐I (12.7 m^2^ g^−1^) is larger than that of Fe_3_O_4_/C (2.4 m^2^ g^−1^) due to the drastically reduced size of Fe_3_O_4_ nanoparticles and the presence of graphene. By comparison with Fe_3_O_4_/C@G‐I, the specific surface area of Fe_3_O_4_/C@G‐II decreased substantially to 3.1 m^2^ g^−1^ after increasing the iron (III) 2‐ethylhexanoate content, which is because of the increased amount of Fe_3_O_4_/C on the graphene basal plane.

### 2.2 Electrochemical Characterizations for SIBs

**Figure 3:**
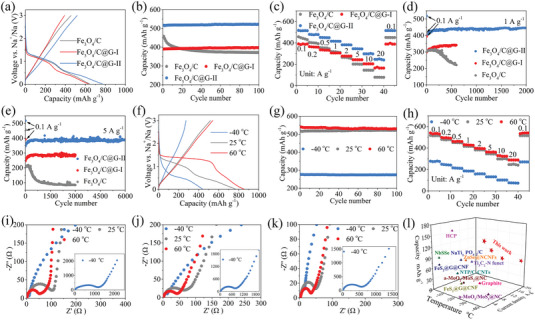
Electrochemical performance characterizations. a–e) Comparison of the electrochemical performance of the Fe_3_O_4_/C, Fe_3_O_4_/C@G‐I, and Fe_3_O_4_/C@G‐II electrodes at 25 °C: a) Voltage profiles of the first cycle at 0.1 A g^−1^, b) cycling performance at 0.1 A g^−1^, c) rate performance, and d,e) long‐term cycling performance at d) 1 A g^−1^ and e) 5 A g^−1^. f–l) Electrochemical performance of the Fe_3_O_4_/C@G‐II electrodes at −40, 25, and 60 °C: f) Voltage profiles of the first cycle at 0.1 A g^−1^, g) cycling performance at 0.1 A g^−1^, h) rate performance, i,j) Nyquist plots before i) and after 1 cycle j) and after rate test k). l) Rate capability comparison of Fe_3_O_4_/C@G‐II with previously reported anodes for SIBs at low operating temperatures.

**Figure 4:**
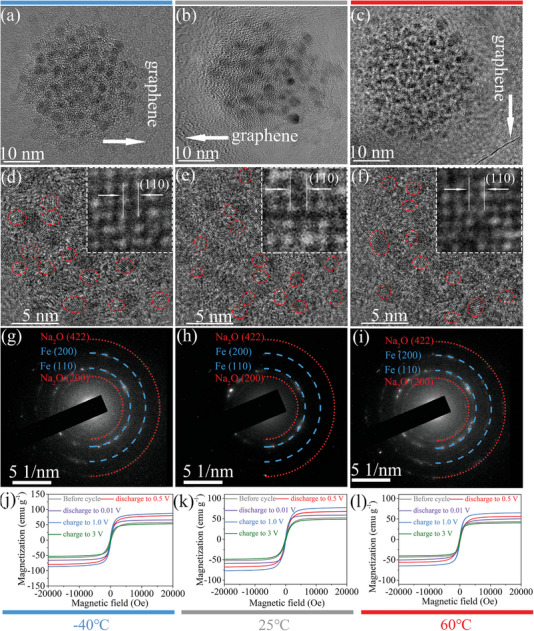
Electrode structure and magnetic response for cycled Fe_3_O_4_/C@G‐II electrode at −40, 25, and 60 °C. TEM and HRTEM images of Fe_3_O_4_/C@G‐II electrode after discharging to 0.01 V at −40 °C a,d), 25 °C b,e), and 60 °C c,f). SAED patterns of Fe_3_O_4_/C@G‐II electrode after discharging to 0.01 V at g) −40 °C, h) 25 °C, and i) 60 °C. Magnetic hysteresis loops of Fe_3_O_4_/C@G‐II electrode during discharge/charge at j) −40 °C, k) 25 °C, and l) 60 °C.

**Figure 5:**
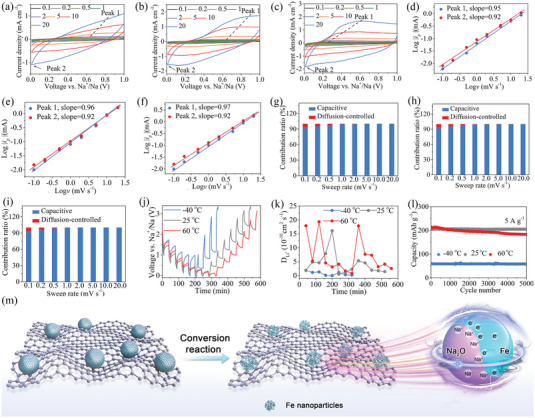
Kinetic analysis of Fe_3_O_4_/C@G‐II electrode at −40, 25, and 60°C in the potential range of 0.01‐1.0 V: CV curves at −40°C a), 25°C b), and 60°C c). Log *i_p_* against Log *v* at marked peaks at −40°C d), 25°C e), and 60°C f). The percentages of pseudocapacitive contribution at different sweep rates at −40°C g), 25°C h), and 60°C i). j) Potential response and k) *D_Na+_* variation of Fe_3_O_4_/C@G‐II electrode during GITT measurement. l) Cycling performance of Fe_3_O_4_/C@G‐II electrode at −40, 25, and 60°C in the potential range of 0.01‐1.0 V. m) Schematic illustration of structural change during coversion reaction and the formation of a space charge zone in the surface capacitance model for extra Na^+^ storage.

The electrochemical performances of the prepared electrodes in half‐cells are exhibited in **Figure** **3**. Figure 3a shows the charging/discharging profiles of these electrodes at a current density of 0.1 A g^−1^. Fe_3_O_4_/C delivers a first charging capacity of 455.8 mAh g^−1^. The introduction of low‐capacity graphene (≈322 mAh g^−1^, Figure S9a, Supporting Information) leads to a reduction in the proportion of Fe_3_O_4_ thus making the capacity of Fe_3_O_4_/C@G‐I only 390.6 mAh g^−1^. Increasing the amount of Fe_3_O_4_ leads to a significantly improved capacity of 517.1 mAh g^−1^ for Fe_3_O_4_/C@G‐II. The higher capacity of the Fe_3_O_4_/C@G‐II than Fe_3_O_4_/C may be due to the fact that 1) the highly conductive graphene facilitates the transport of sodium ions in the electrode, thus boosting more fully sodiation reaction of Fe_3_O_4_, 2) the reduced Fe_3_O_4_/C size of Fe_3_O_4_/C@G‐II shortens the diffusion distance of sodium ions, which results in a more complete sodiation of Fe_3_O_4_, and 3) the increased interface between Fe_3_O_4_/C and graphene can be used for sodium ion storage, thereby increases the capacity of Fe_3_O_4_/C@G‐II. With the increase of the specific surface area, the gradually decreased initial Coulomb efficiency (ICE) of 70.8% for Fe_3_O_4_/C, 67.1% for Fe_3_O_4_/C@G‐II, and 62.0% for Fe_3_O_4_/C@G‐I are obtained due to the formation of more solid electrolyte interphase (SEI) layers.^[^ ^29^ ^]^ It is apparent that the ICE of these electrodes are relatively low. Notably, the low ICE of Fe_3_O_4_ anodes in SIBs is a pervasive issue.^[^ ^30^, ^31^, ^32^, ^33^ ^]^ This is primarily attributed to: 1) during the initial discharge phase of the Fe_3_O_4_ electrode, the formation of the SEI layers at the Fe_3_O_4_/electrolyte interface results in the substantial consumption of sodium ions, and 2) deep discharge of the Fe_3_O_4_ electrode initiates conversion reactions, leading to severe structural fragmentation and exposing a significant amount of fresh surface to the electrolyte. This exposure contributes to the generation of additional SEI layers, thereby causing a considerably low ICE (≈50%).^[^ ^30^, ^31^, ^32^, ^33^ ^]^ Although the preparation of carbon coatings on the surface of Fe_3_O_4_ particles can, to a certain extent, mitigate the expansion and pulverization of Fe_3_O_4_, these carbon coatings struggle to withstand the significant impact of conversion reactions. The electrode structure remains vulnerable to damage, forming new SEI layers and limiting the enhancement of ICE (≈65%), as displayed in this study and other reports.^[^ ^34^, ^35^, ^36^ ^]^ It is essential to highlight that the low specific surface area and a stable electrode/electrolyte interface are crucial for achieving high ICE, and core‐shell structures with enough internal space and low specific surface area may be promising considerations. In the subsequent 100 cycles, no obvious capacity loss can be detected for Fe_3_O_4_/C@G‐I (102.2%, capacity retention) and Fe_3_O_4_/C@G‐II (101.0%, capacity retention) electrodes (Figure 3b), denoting excellent cycling stability. This can also be confirmed by the free‐crack surface morphology (Figure S10a,b, Supporting Information) and the slightly increased thickness of the electrode (Figure S10c,d, Supporting Information) after cycling. Such excellent cycling stability is mainly due to 1) the nano‐sized Fe_3_O_4_ relieves stress concentrations, 2) graphene effectively carries the Fe_3_O_4_/C and superparamagnetic Fe nanoparticles (after the first discharge, confirmed in **Figure** **4**b) to maintain the overall integrity of the Fe_3_O_4_/C@G, even when subjected to the violent impact of the conversion reaction, and 3) the flexibility and the natural fold space of graphene accommodate the volume expansion of the electrodes. In contrast, the Fe_3_O_4_/C electrode shows poor cycling stability due to its large size and the lack of graphene. The significantly increased thickness of the electrode after cycling further reveals the reason for the bad cycling stability of Fe_3_O_4_/C (Figure S11, Supporting Information). Figure 3c exhibits the rate performance of these electrodes. At 2 A g^−1^, the capacity of Fe_3_O_4_/C@G‐II electrode maintains at 381.9 mAh g^−1^, corresponding to 74.1% of the capacity at 0.1 A g^−1^ (515.2 mAh g^−1^). Even after cycling at 10 and 20 A g^−1^, Fe_3_O_4_/C@G‐II electrode still shows high capacities of 298.1 and 245.1 mAh g^−1^, respectively. Furthermore, the capacity is absolutely restored when the current density returns to 0.1 A g^−1^ again. In contrast, Fe_3_O_4_/C and Fe_3_O_4_/C@G‐I electrodes display far inferior rate capacity than Fe_3_O_4_/C@G‐II. Note that graphene in Fe_3_O_4_/C@G electrodes mainly serves as a carrier and provides an efficient electron transport platform, and does not provide strong rate capacity (Figure S9b, Supporting Information). Such superior rate capacity of Fe_3_O_4_/C@G‐II is largely due to its strong charge transport capability confirmed by the electrochemical impedance spectroscopy (EIS) study (detailed discussion in Figure S2 Supporting Information), and strong capacitance contribution, and high sodium ion diffusion coefficient (*D*_Na+_) evidenced by the cyclic voltammetry (CV) tests (detailed discussion in Figures S13–S15 in Supporting Information). The long‐term cycling stability of the Fe_3_O_4_/C, Fe_3_O_4_/C@G‐I, and Fe_3_O_4_/C@G‐II electrodes is also tested. When testing at 1 A g^−1^, the Fe_3_O_4_/C@G‐II electrode delivers a reversible capacity of 445.2 mAh g^−1^ after 2000 cycles without any capacity loss (Figure 3d). Even after 6000 cycles at 5 A g^−1^, the Fe_3_O_4_/C@G‐II electrode still shows high reversible capacity and cycle stability, achieving 386.0 mAh g^−1^ with a considerable capacity retention of 111.7% (Figure 3e). The initial increasing specific capacity during 1–600 cycles may be due to the excess interfacial sodium storage and the activation of the electrode.^[^ ^30^ ^]^ In comparison, Fe_3_O_4_/C@G‐I exhibits relatively lower capacity at 1 and 5 A g^−1^ due to its lower Fe_3_O_4_ content. Furthermore, during 600 cycles at 1 A g^−1^ (Figure 3d) and 3000 cycles at 5 A g^−1^ (Figure 3e), the Fe_3_O_4_/C@G‐I electrode displays cycling stability similar to that of the Fe_3_O_4_/C@G‐II electrode. Conversely, the Fe_3_O_4_/C electrode experiences rapid capacity decay at both 1 A g^−1^ (Figure 3d) and 5 A g^−1^ (Figure 3e). The long cycling results further affirm the superior electrochemical performance of the Fe_3_O_4_/C@G‐II electrode compared to Fe_3_O_4_/C@G‐I and Fe_3_O_4_/C electrodes. Based on the reaction system for the synthesis of Fe_3_O_4_/C@G‐II, we further increase the amount of iron (III) 2‐ethylhexanoate to obtain Fe_3_O_4_/C@G‐III. In contrast to the uniform nanoscale structure of Fe_3_O_4_/C@G‐II, Fe_3_O_4_/C@G‐III exhibits a distinct microscale block‐like morphology and substantial graphene aggregation (Figure S16, Supporting Information). Furthermore, the initial charge capacity (487.9 mAh g^−1^), cycling stability (87.8%, capacity retention, 100 cycles, 0.1 A g^−1^), and rate performance (212.6 mAh g^−1^, 10 A g^−1^; 161.0 mAh g^−1^, 20 A g^−1^) of Fe_3_O_4_/C@G‐III are all inferior to those of Fe_3_O_4_/C@G‐II (Figure S17, Supporting Information). This suggests that an excess of iron (III) 2‐ethylhexanoate is detrimental to the construction of nanostructures and Na‐ion storage performance. This may be attributed to 1) the microscale dimensions of Fe_3_O_4_/C@G‐III, which significantly extend the diffusion path for sodium ions, resulting in reduced sodiation efficiency of Fe_3_O_4_ and, as a result, lower electrode capacity and compromised rate performance. Furthermore, the microscale size contributes to severe electrode pulverization, diminishing cycling stability; 2) graphene aggregation, which decreases the interface between Fe_3_O_4_/C and graphene, impeding additional sodium ion storage. Additionally, aggregated graphene hinders electron transport, preventing complete sodiation reaction, ultimately leading to reduced electrode capacity and compromised rate performance.

Given the excellent electrochemical performances of the Fe_3_O_4_/C@G‐II electrode, further wide operation‐temperature electrochemical performance tests are carried out on this electrode (Figure 3f–l). Compared to the first charging capacity of 517.1 mAh g^−1^ delivered at 25 °C (Figure 3f), the slightly higher capacity (540.4 mAh g^−1^) at 60 °C is mainly because high temperatures reduce the Na‐ion diffusion barriers thus improving the Na^+^ storage.^[^ ^38^ ^]^ In general, severe capacity loss occurs at low temperatures due to the increase of energy barrier of Na‐ion diffusion.^[^ ^39^ ^]^ Despite this, the Fe_3_O_4_/C@G‐II electrode still delivers a competitive capacity of 273.7 mAh g^−1^ (53% that at 25 °C) at an ultra‐low temperature of −40 °C. Furthermore, as the operating temperature decreases, an increase in polarization and a decline in voltage plateau are observed (Figure 3f). This is primarily attributed to the elevated viscosity of the electrolyte and the increased impedance at the electrode/electrolyte interface at lower temperatures, resulting in reduced ionic conductivity and, consequently, heightened polarization and a lowered discharge voltage plateau.^[^ ^40^, ^41^ ^]^ Additionally, the Fe_3_O_4_/C@G‐II electrode operated at 60 °C exhibits a relatively lower capacity retention (98.1%) during the subsequent 100 cycles compared to the electrodes operated at 25 and −40 °C, where the capacity retention is ≈100% (Figure 3g). This can be attributed to the heightened occurrence of interfacial side reactions at elevated operating temperatures.^[^ ^40^ ^]^ Moreover, to validate the source of electrode capacity at low temperatures, we remove the Fe_3_O_4_ phase from Fe_3_O_4_/C@G‐II through etching with HCl. The XRD spectrum shows that Fe_3_O_4_ in Fe_3_O_4_/C@G‐II has been completely removed after etching (Figure S18, Supporting Information). In Figure S19 (Supporting Information), the etched Fe_3_O_4_/C@G‐II exhibits an initial charge capacity of 83.5 mAh g^−1^ at −40 °C and a current density of 0.1 A g^−1^, significantly lower than the capacity of Fe_3_O_4_/C@G‐II under the same testing conditions. This suggests that the electrode capacity of Fe_3_O_4_/C@G‐II at low temperatures predominantly arises from Fe_3_O_4_ rather than the carbon nanolayers and graphene. Figure 3h shows the rate performance of Fe_3_O_4_/C@G‐II electrodes at different temperatures. The rate capacities at 60 °C are slightly higher than that at 25 °C throughout the rate testing, which is attributed to accelerated electron/ion transfer at high temperatures.^[^ ^42^ ^]^ When testing at −40 °C, a satisfactory rate capability is still obtained. Specifically, the capacity maintains 158.6 mAh g^−1^ at 2 A g^−1^ (−40° C), corresponding to 41.5% that at 25 °C (381.9 mAh g^−1^). Even testing at 10 and 20 A g^−1^, the electrode still delivers capacities of 101.3 and 74.0 mAh g^−1^, respectively, corresponding to 30.2% and 25.7% of those at 25 °C. Moreover, capacity can be restored when the current density returns to 0.1 A g^−1^ again.

We carry out the EIS study to reveal the charge transport capability of Fe_3_O_4_/C@G‐II under wide operation‐temperature conditions (Figure 3i–k). The diameter of the semicircle corresponds to the charge transfer resistance (*R*_ct_) and the slope of the sloping line relates to the Warburg impedance (W) of Na^+^ diffusion.^[^ ^43^ ^]^ Before cycling, the *R*_ct_ increases with the decrease in the operating temperature (Figure 3i). During cycling, the *R*_ct_ continues to decrease at different temperatures (Figure 3j,k), typically due to the electrodes being activated and stabilizing during the cycle.^[^ ^44^ ^]^ In addition, the slope of the sloping line of the Fe_3_O_4_/C@G‐II electrode at 25 and 60 °C is basically the same, which is larger than that at −40 °C. These results demonstrate that the sodium ion diffusion capability of Fe_3_O_4_/C@G‐II weakens as the operating temperature decreases, in agreement with the rate performances in Figure 3h. Even though Fe_3_O_4_/C@G‐II experiences a reduction in rate performance at an extraordinarily low operating temperature of −40 °C, it continues to outperform the majority of previously reported SIB anode materials in terms of low‐temperature rate capability (Figure 3l; Table S1, Supporting Information). Moreover, when compared to previously reported SIB anodes, Fe_3_O_4_/C@G‐II exhibits remarkable competitiveness in cycling stability at room temperature and elevated operating temperatures (Tables S2 and S3, Supporting Information).

To understand the high capacity and excellent rate capability of Fe_3_O_4_/C@G‐II electrode over a wide temperature range, it is necessary to start with its unique conversion‐type Na^+^ storage mechanism. Figure 4a–c shows the morphology of Fe_3_O_4_/C@G‐II electrode after discharging to 0.01 V at −40, 25, and 60 °C, respectively. Apparently, Fe_3_O_4_/C nanoparticles suffer severe structural damage during the conversion reaction at these operating temperatures, but thanks to the presence of graphene, the reaction products can still be loaded on the graphene basal plane and maintain efficient electron transport. In the HRTEM images (Figure 4d–f), numerous nanoparticles with an average size of ≈2 nm can be obtained. A distinct lattice fringe with a d‐spacing of about 0.20 nm can be observed at temperatures of −40, 25, and 60 °C (insets of Figure 4d–f), which corresponds to the (110) crystal plane of metallic Fe^0^. SAED patterns reveal the complete disappearance of diffraction spots and rings ([111], [220], [311], [422], and [620], Figure 1d) after the initial discharge to 0.01 V at −40 (Figure 4g), 25 (Figure 4h), and 60 °C (Figure 4i), respectively, which typically represent polycrystalline Fe_3_O_4_. Instead, distinct diffraction rings of (110), (200) planes of Fe (JCPDS: 06–0696) and (200), (422) planes of Na_2_O (JCPDS: 03–1074) emerge. Correspondingly, Fe_3_O_4_ nanoparticles with an initial size of ≈80 nm undergo a transformation into Fe^0^ nanoparticles with a reduced size of around 2 nm. Besides, the microstructure and the SAED patterns of electrodes after fully discharging at different temperatures remain consistently similar. These results suggest that within the temperature range of −40 to 60 °C, the Fe_3_O_4_ phase in the Fe_3_O_4_/C@G‐II electrode undergoes complete transformation into Fe^0^ and Na_2_O phases after the initial sodiation. Furthermore, Figures S20–S22 (Supporting Information) show that these ultra‐tiny Fe^0^ nanoparticles (white dots in HAADF images) generated at −40, 25, and 60 °C are uniformly dispersed in the matrix. The structural evolution of the Fe_3_O_4_/C@G‐II electrode during subsequent cycles within an operating temperature range of −40–60 °C is further monitored. It can be observed that the microstructure structure of the Fe_3_O_4_/C@G‐II electrode, operating at −40 °C (Figures S23 and S24, Supporting Information), 25 °C (Figures S25 and S26, Supporting Information), and 60 °C (Figures S27 and S28, Supporting Information), remains largely unchanged during the process of recharging to 3 V and subsequently discharging to 0.01 V. Additionally, throughout the charge–discharge process, no notable changes are observed in the size of the nanoparticles on the graphene plane. Furthermore, even after 100 cycles −40 °C (Figure S29, Supporting Information), 25 °C (Figure S30, Supporting Information), and 60 °C (Figure S31, Supporting Information) and at 0.1 A g^−1^, these ultra‐small nanoparticles remain stably loaded on the graphene basal planes, exhibiting a similar morphology with their initial appearance after the first discharge to 0.01 V. This further substantiates the role of graphene as a carrier for these nanocrystals, ensuring the electrode's stable performance despite structural disruptions caused by conversion reactions. It can be concluded that the conversion‐type sodium storage mechanism of Fe_3_O_4_/C@G‐II electrode can function properly in the operating temperature range of −40–60 °C. Compared to Fe_3_O_4_/C@G‐II electrode, Fe_3_O_4_/C electrode produce many Fe^0^ nanoparticles of ≈5 nm in size (Figures S32 and S33, Supporting Information), which is more than twice the size of Fe^0^ nanoparticles in Fe_3_O_4_/C@G‐II electrodes. The large size difference of the Fe^0^ nanoparticles in both electrodes is most likely related to the time that the conversion reaction undergoes. For Fe_3_O_4_/C@G‐II electrode, the ultra‐high conductivity of graphene provides efficient electron transport for the conversion reaction, which allows the conversion reaction to proceed so quickly that the Fe^0^ nuclei do not have enough time to grow into large particles. For Fe_3_O_4_/C electrode, the structural collapse caused by the conversion reaction severely damages the electron transport network of the electrode, blocking the conversion reaction, and giving the Fe^0^ nuclei enough time to grow into large particles during the slow conversion reaction. Besides, Fe_3_O_4_/C@G‐II electrode has a smaller size (≈80 nm) of Fe_3_O_4_ nanoparticles than ≈150 nm of Fe_3_O_4_/C, which can shorten sodium ion diffusion distance and thus also results in a faster conversion reaction. The higher surface‐to‐volume ratio of Fe^0^ nanoparticles in Fe_3_O_4_/C@G‐II electrode provides a solid foundation for achieving a stronger surface capacitance effect, which is confirmed by the stronger capacitance contribution of Fe_3_O_4_/C@G‐II electrode (Figure S13g–i, Supporting Information) than Fe_3_O_4_/C electrode (Figure S13a–c, Supporting Information). Notably, the ultra‐tiny Fe^0^ nanoparticles generated from the Fe_3_O_4_/C@G‐II electrode can construct space charge zone for Na^+^ storage based on the spin‐polarized surface‐capacitance effect.^[^ ^45^ ^]^ Fe, as a typical transition metal, is not full of electrons in its 3d orbitals. Once ultra‐tiny Fe^0^ nanoparticles are generated during the conversion reaction, a large number of spin‐polarized electrons are injected into their 3d orbits under the action of an electric field. Meanwhile, sodium ions are stored in the formed Na_2_O matrix. Spin‐polarized electrons and Na^+^ are mutually attracted, leading to their accumulation at the Fe^0^/Na_2_O interface to create a space charge zone, thereby inducing spin‐polarized surface capacitance to store extra Na^+^.^[^ ^45^, ^46^ ^]^ In contrast to traditional electrode materials where ions and electrons must coexist in the same phase for transport, the constructed space charge zone in the Fe_3_O_4_/C@G‐II electrode significantly extends the space. This allows Na‐ion transport and storage to occur within one phase (Na_2_O), while electron transport and modulation take place within another phase (Fe^0^ nanoparticles), thereby imparting rapid charge transfer capability to the electrode. Because magnetic properties are extremely sensitive to phase constitution, electron transfer, and structural variations of transition metals and their compounds,^[^ ^47^ ^]^ we further study the Na‐ion storage mechanism of Fe_3_O_4_/C@G‐II electrode by hysteresis curves, which shows superparamagnetic behavior due to the formation of ultra‐small Fe^0^ nanoparticles. Obvious anodic and cathodic peaks are detected for Fe_3_O_4_/C@G‐II electrode tested at −40 °C (1.53 and 0.61 V, Figure S34, Supporting Information), 25 °C (1.56 and 0.84 V, Figure S35, Supporting Information), and 60 °C (1.67 and 0.91 V, Figure S36, Supporting Information). This means that the potentials at which the reduction reaction occurs at operating temperatures of −40–60 °C are all higher than 0.5 V, i.e., the Fe^0^ nanoparticles have been fully transformed when the electrode is discharged to 0.5 V. At the same time, the oxidation potentials of the electrodes operating at these temperatures are all higher than 1 V, indicating that Fe elements still exist in the form of Fe^0^ nanoparticles (unoxidized) when charged to 1 V. For the pristine electrode, the magnetization at −40, 25, and 60 °C are 57.5 (Figure 4j), 52.1(Figure 4k), and 43.6 emu g^−1^ (Figure 4l), respectively. The difference in magnetization is due to the magnetic moment significantly suppressed by temperature.^[^ ^48^ ^]^ In the first discharge from the open‐circuit voltage to 0.5 V, the conversion reaction induces a considerable increase in magnetization (from 57.5 to 79.7 emu g^−1^, −40 °C; from 52.1 to 68.2 emu g^−1^, 25 °C; from 43.6 to 56.0 emu g^−1^, 60 °C), which is due to the formation of superparamagnetic Fe nanoparticles. Upon further discharge, the magnetization then reduces to values of 65.7 emu g^−1^ for −40 °C, 59.2 emu g^−1^ for 25 °C, and 56.3 emu g^−1^ for 60 °C at the terminal discharge potential of 0.01 V. The magnetization change can be explained as follows: superparamagnetic Fe^0^ nanoparticles and Na_2_O matrix can be worked as the electron and Na^+^ accepting phases, respectively. Charge separation occurs at the Fe/Na_2_O interface, which is referred to as a “job‐sharing” mechanism that offers additional capacity to the cell.^[^ ^49^ ^]^ In addition, the extra electrons stored in Fe partially offset the spin majority band at the 3d energy level, leading to a reduction in magnetization. This result indicates that the fully reduced superparamagnetic Fe^0^ nanoparticles can still be involved in Na^+^ storage and thus decrease the magnetization of the electrode. Interestingly, the following charging up to 1.0 V leads to a significant increase in the magnetization (to 87.6 emu g^−1^, −40 °C; to 76.8 emu g^−1^, 25 °C; to 64.7 emu g^−1^, 60 °C). Further charging leads to a drastic reduction in the magnetization (to 53.2 emu g^−1^, −40 °C; to 48.4 emu g^−1^, 25 °C; to 40.3 emu g^−1^, 60 °C) because of the oxidation of the metallic Fe. These results indicate that Na^+^ can be stored on the Fe surface to fabricate the space charge zone over a wide operating temperature range and confirm the strong surface‐capacitance effect of Fe^0^ nanoparticles. This is the first time that the spin‐polarized surface capacitance effect of Fe_3_O_4_ anodes has been observed under ultra‐low and high operating temperatures, which provides fast Na‐ion transport and extra sodium storage for wide operation‐temperature SIBs.

To further study the kinetic property of the Fe_3_O_4_/C@G‐II electrode about the spin‐polarized surface capacitance effect, we perform CV tests at different scan rates in a lower potential range of 0.01–1.0 V. Due to the presence of the space charge region, rectangular CV profiles representing capacitive behavior occur in the voltage range of 0.01–1.0 V for the Fe_3_O_4_/C@G‐II electrode tested at −40, 25, and 60 °C (**Figure** **5**a–c). For further insight, we plot log |*i*| as a function of log *υ*, where *i* and *υ* correspond to the current and scan rate, respectively. The relationship between the current (*i*) and the scan rate (*v*) obeys Equation (1).^[^ ^50^, ^51^, ^52^ ^]^

(1) $$i=av^{b}$$

where a and b are empirical constants. It is found that the *b*‐values of both anodic and cathodic scanning peaks are approximately equal to 1 for Fe_3_O_4_/C@G‐II electrode operated at −40, 25, and 60 °C over the scan rate range of 0.1 to 20 mV s^−1^ (Figure 5d–f), which suggests that a strong capacitive response occurs on the Fe_3_O_4_/C@G‐II electrode over a wide operation temperature range. Moreover, the percentage capacitive contribution of the Fe_3_O_4_/C@G‐II electrode operated at different temperatures (−40, 25, and 60 °C) exhibits increasing trends as the scan rate increased from 0.1 to 20.0 mV s^−1^ (from 93.80 to 99.99%, −40°C, Figure 5g; from 94.02 to 99.99%, 25°C, Figure 5h; from 94.24 to 99.99%, 60°C, Figure 5i). Such high capacitive contribution in the potential range of 0.01–1.0 V can be addressed to the formation of the space charge zone, which is beneficial for obtaining high capacity and excellent rate capability.

To directly demonstrate the high efficiency of Na‐ion transport, we use the galvanostatic intermittent titration technique (GITT) to calculate *D*_Na+_ of Fe_3_O_4_/C@G‐II electrode operating at −40, 25, and 60 °C. *D*_Na+_ in the GITT obeys Fick's second law, as in Equation (2).^[^ ^53^, ^54^, ^55^ ^]^

(2) $$D=\frac{4}{\pi \tau }{\left(\frac{m_{B}V_{M}}{M_{B}S}\right)}^{2}\left(\frac{\Delta E_{S}}{\Delta E_{\tau }}\right)$$

where *m_B_*, *m_B_*, and *Vm* represent the molar mass, mass, and molar volume of the active material, respectively. *S*, *τ*, *ΔE_S_*, and *ΔE_τ_* are electrode area, constant current time, and voltage change, as well as total voltage change, respectively. The values of *τ*, *ΔE*_S_, and *ΔE*_τ_ are determined from the GITT profiles (Figure 5j). According to Equation (2), the *D*_Na+_ of the Fe_3_O_4_/C@G‐II electrode at −40, 25, and 60 °C can be calculated to be 1.0 × 10^−13^−1.87 × 10^−10^, 5.6 × 10^−11^−1.61 × 10^−9^, and 8.3 × 10^−11^−1.93 × 10^−9^ cm^2^ s^−1^, respectively (Figure 5k). This result indicates that spin‐polarized surface capacitance effects significantly boost Na‐ion transport in Fe_3_O_4_/C@G‐II electrode, even at the ultra‐low operating temperature of −40 °C. To demonstrate the reliability of the spin‐polarized surface capacitance effects, we perform the long‐term cycling tests on Fe_3_O_4_/C@G‐II electrode at −40, 25, and 60 °C in the potential range of 0.01–1.0 V (Figure 5l). At 5 A g^−1^, the Fe_3_O_4_/C@G‐II electrode shows reversible capacities of 61.2, 207.2, and 215.2 mAh g^−1^ at −40, 25, and 60 °C, respectively. Note that the capacities in the potential range of 0.01–1.0 V are 46.6, 60.1, and 59.4% of those at −40, 25, and 60 °C in the potential range of 0.01–3.0 V, respectively. This result indicates the spin‐polarized surface capacitance effects contribute significantly to the capacity of the Fe_3_O_4_/C@G‐II electrode. After 5000 cycles, high capacity retentions of 95.3%, 98.9%, and 85.3% are obtained for the electrode tested at −40, 25, and 60 °C, respectively, confirming the high reliability of the spin‐polarized surface capacitance effects. Furthermore, the capacity retention of the electrode operating at 60 °C is notably lower than that of the electrodes operating at 25 and −40 °C. In fact, this is a common phenomenon in wide‐temperature‐range SIBs, and similar occurrences have been frequently reported in previous studies.^[^ ^40^, ^56^ ^]^ This is primarily attributed to the acceleration of electrolyte decomposition at elevated temperatures, initiating irreversible side reactions that compromise the cycling stability of the battery.^[^ ^40^, ^56^ ^]^ According to the above results, the schematic illustration of structural change during conversion reaction and the formation of a space charge zone for extra Na^+^ storage is shown in Figure 5m. The electrode structure after conversion reaction, magnetic hysteresis loops, and CV testing results of Fe_3_O_4_/C@G‐II electrode strongly support that the spin‐polarized surface capacitance effects endow Fe_3_O_4_/C@G‐II electrode with rapid Na‐ion transport and high capacity for SIBs over a wide operation temperature range.

### 2.3 Electrochemical Characterizations of Full Cells in SIBs

**Figure 6:**
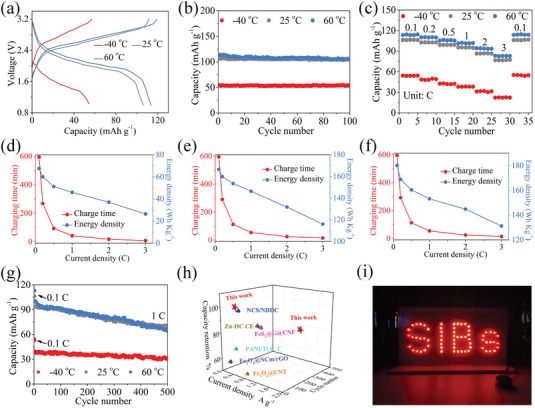
Full cells testing and comparison. a) Galvanostatic charge/discharge voltage curves at 0.1C, b) cycling performances at 0.1C, c) rate performances of the full cell at 25, 60, and −40 °C. Relationship of charging time and energy density of the full cell at d) 25 °C, e) 60 °C, and f) −40 °C. g) long‐term cycling performances of the full cell at 1C at 25, 60, and −40 °C. h) Cycle stability comparison with recently reported anode materials for SIBs. i) Optical image of LED arrays powered by the full cell.

As a practical demonstration, Fe_3_O_4_/C@G‐II is utilized as an anode coupling with Na_3_V_2_(PO_4_)_3_ cathode to assemble the full cell to assess the battery performance under wide operation‐temperature range. Notably, the full cell delivers high discharge capacities of 113.5, 106.1, and 54.0 mAh g^−1^ at 60, 25, and −40 °C, respectively (**Figure** **6**a). No obvious capacity decay can be found after 100 cycles at 0.1C (Figure 6b), signaling excellent cycling stability of the full cell under wide operation‐temperature range. Figure 6c exhibits the rate performance, as can be seen, the rate capacities of the full cell at 60 °C are slightly higher than that at 25 °C, which is similar to the results in the half‐cell. Significantly, the full cell still shows good rate performance despite extremely harsh low‐temperature conditions (−40 °C), such as 39.0mAh g^−1^ for 1C (40.1% that of 25 °C) and 22.4 mAh g^−1^ for 3C (30.2% that of 25 °C). We further calculate the gravimetric energy densities of the full cell at −40, 25, and 60 °C based on the equation that gravimetric energy density (Wh kg^−1^) = (Cc × V)/*m*_active_, Cc:cell capacity (mAh); V: nominal voltage (V); *m*_active_: active mass of cathode and anode (mg). The calculated energy densities at −40, 25, and 60 °C are 67.6, 166.4, and 180.1 Wh kg^−1^, respectively. At −40 °C, the energy density can reach 26.5 Wh kg^−1^ after charging for 8.4 min at 3C (Figure 6d). Raising the operating temperature to 25 °C, the energy density can reach 115.9 Wh kg^−1^ after charging for 14.5 min at 3C, which is 72.6% of the energy density at 0.1C (Figure 6e). When operating at 60 °C, the energy density can be as high as 131.2 Wh kg^−1^ with the same current density/charging time as at 25 °C (Figure 6f), denoting the excellent fast‐charging capability of Fe_3_O_4_/C@G‐II under a wide operation‐temperature range. Moreover, we also perform long‐term cycling to demonstrate the reliability of the full cell. After 500 cycles at 1C, the capacity retention at −40, 25, and 60 °C is 77.2, 75.7, and 65.2%, respectively (Figure 6g). It is clear that capacity retention increases as the operating temperature decreases, possibly due to slower electrolyte degradation at lower temperatures. These results fully confirm the excellent fast‐charging capability and long cycle life of Fe_3_O_4_/C@G‐II as an anode for wide‐temperature SIBs. When compared with recently reported anode materials for SIBs, the full cell also shows obvious advantages in terms of cycling stability (Figure 6h; Table S4, Supporting Information). Furthermore, the LED arrays can operate successfully using the assembled full cell (Figure 6i). These results demonstrate the huge potential of Fe_3_O_4_/C@G‐II for practical applications.

## 3 Conclusion

In summary, the Fe_3_O_4_ nanoparticles encapsulated by carbon nanolayers that are uniformly dispersed on the graphene basal plane are prepared via a one‐step pressure‐induced vapor synthetic process and successfully utilized as anode materials for SIBs over a wide operation temperature range. It is revealed that superparamagnetic Fe nanoparticles generated from Fe_3_O_4_ in conversion reactions can induce strong surface‐capacitance effects in the potential of 0.01–1 V to boost Na‐ion transport and storage at operating temperatures ranging from −40 to 60 °C. The introduction of graphene reduces the size of the superparamagnetic Fe nanoparticles, enhancing the spin‐polarized surface capacitance effect. With strong spin‐polarized surface capacitance effect and rational structure, the Fe_3_O_4_/C@G‐II delivers high specific capacity, excellent rate capability, and long cycling life in half cells at a wide operating temperature range of −40–60 °C. The assembled full cell also shows superior fast‐charging capability and cycling stability at operating temperatures of −40–60 °C. This work may provide a boost to the development of Fe_3_O_4_‐based anodes in wide‐temperature‐range SIBs.

## Conflict of Interest

The authors declare no conflict of interest.

## Supporting information

Supporting Information

## Data Availability

The data that support the findings of this study are available from the corresponding author upon reasonable request.
