# Supplementary material for: Spin‐Polarized Surface Capacitance Effects Enable Fe3O4 Anode Superior Wide Operation‐Temperature Sodium Storage
Source: Adv Sci (Weinh). 2023 Dec 7;11(6):2306992. doi: 10.1002/advs.202306992 (PMC10853739; doi:10.1002/advs.202306992)
Supplement: Supplementary file 1 — Supporting Information [file ADVS-11-2306992-s001.pdf]

ADVANCED SCIENCE
Open Access

Supporting Information

for *Adv. Sci.*, DOI 10.1002/advs.202306992

Spin-Polarized Surface Capacitance Effects Enable Fe_3O_4 Anode Superior Wide Operation-Temperature Sodium Storage

Zhenwei Li, Meisheng Han, Peilun Yu and Jie Yu**

Supporting Information

Spin-Polarized Surface Capacitance Effects Enable Fe_3O_4 Anode Superior Wide Operation-Temperature Sodium Storage

Zhenwei Li[1a,b], Meisheng Han[1c]*, Peilun Yu[a], and Jie Yu[a,b]*

[a]Guangdong Provincial Key Laboratory of Semiconductor Optoelectronic Materials and Intelligent Photonic Systems, Shenzhen Engineering Lab for Supercapacitor Materials, School of Material Science and Engineering, Harbin Institute of Technology, Shenzhen, University Town, Shenzhen 518055, China

[b]Songshan Lake Materials Laboratory Dongguan, Guangdong 523808, China

[c]Department of Mechanical and Energy Engineering, Southern University of Science and Technology, Shenzhen 518055, China

*Corresponding Author.

E-mail address: jyu@hit.edu.cn (J. Yu); hanms@sustech.edu.cn

[1]Both authors contributed equally to this work.

Experimental Section

Preparation of samples: The distinct amount of Iron(III) 2-ethylhexanoate/graphene (1.5 ml/0 mg, Fe_3O_4/C; 1.5 ml/10 mg, Fe_3O_4/C@G-I; 3 ml/10 mg, Fe_3O_4/C@G-II; and 6 ml/10 mg, Fe_3O_4/C@G-III) was evenly mixed in the self-made vessels. These vessels were then sealed in an Ar-filled glove box and subsequently transferred to a tube furnace, where the temperature was raised to 500 °C at a ramp rate of 10 °C/min and held in Ar flow for 30 min. After that, cooled to ambient temperature to obtain these samples. All chemical reagents were purchased from Macklin.

Characterizations: SEM (Hitachi S-4700), TEM (FEI Talos F200x), HRTEM (FEI Talos F200x), Raman spectroscopy (Renishaw RM-1000), XRD (D/max-2500/PC, Rigaku), and XPS (Thermo Scientific Escalab 250Xi), as well as TGA (Pyris I, PerkinElmer), were utilized to measure the chemical composition and microstructure of samples. The surface area, pore volume, and pore size distribution of samples were measured by the BET (ASAP 2020, HD88). The physical property measurement system (PPMS, Quantum Design) was used to test the magnetic hysteresis curves.

Electrochemical measurements: In half cells, the electrodes were fabricated by mixing the active materials, acetylene black, and PVDF with 8:1:1 in NMP solvent, which was then uniformly coated on the copper foil and dried at 110 ℃ for 12 h under vacuum. The mass loading of active materials is about 1.1 mg cm^{-2}. 2032 coin-type cells were assembled in an Ar-filled glove box using sodium foil as the counter/reference electrode, and using Whatman glass fiber as separator absorbing electrolyte (1 M $NaPF_6$ dissloved in diglyme was used as the electrolyte). In full cells,

cathode electrodes composed of $Na_3V_2(PO_4)_3$ (NVP, 80 wt%), PVDF (10 wt%), and acetylene black (10 wt%) were dried under vacuum at 110 ^oC for 12 h. The active mass loading of Fe_3O_4/C@G-II and NVP is about 1.1 and 4.9 mg cm^{-2}, corresponding areal capacity of 0.57 and 0.52 mAh cm^{-2}, respectively, resulting in a N/P ratio of 1.1. Before assembling full cells, the anode is electrochemically activated for five cycles at 0.1 C to enhance its first CE. The battery performances were recorded on the Land CT2001A battery-test system (Wuhan Land Electronic co., China) at the charge/discharge current density of 0.1-20 A g^{-1} between 0.01 and 3 V in half cells, and between 1.0 and 3.2 V in full cells. A CHI 760D electrochemical workstation (Shanghai CH Instruments Co., China) was employed to measure CV and EIS. CV curves were carried out at scanning rates of 0.1-20 mV s^{-1} from 0.01 to 3 V (vs.Na/Na^+). EIS was performed from 10^5 to 10^{-2} Hz with an amplitude of 5 mV. All cells were tested at room temperature.

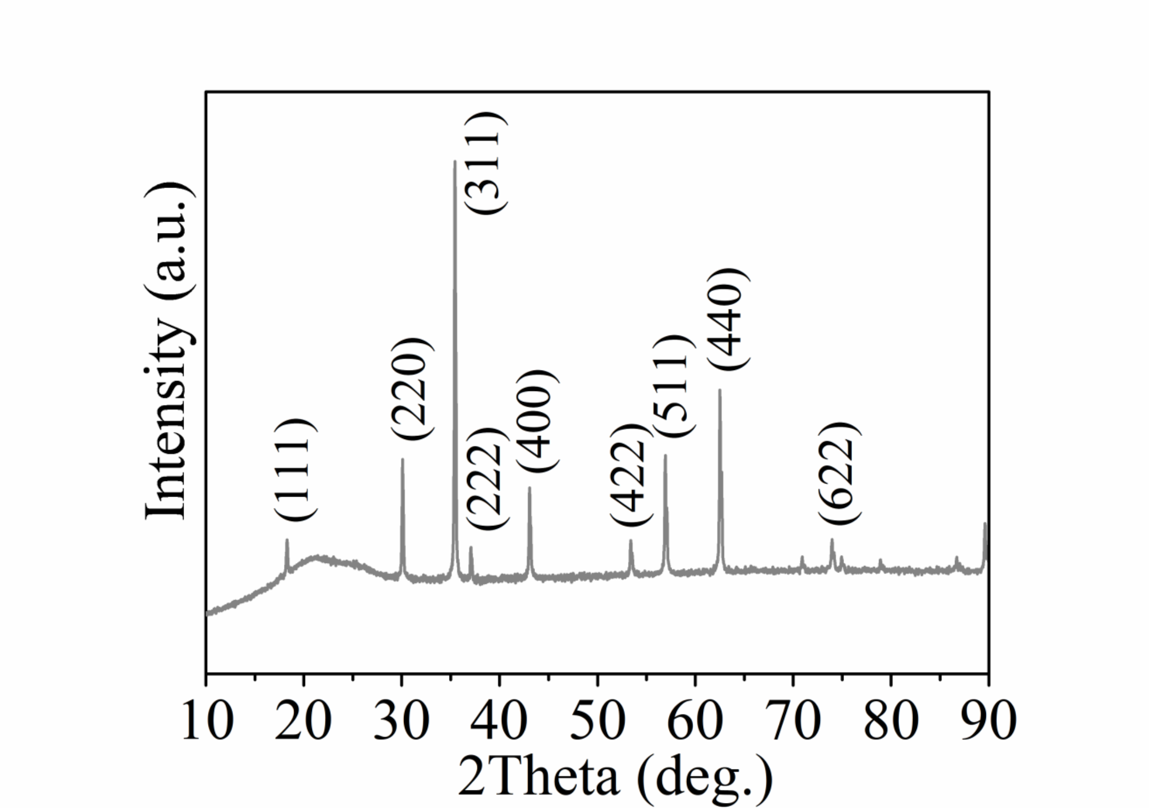

Figure S1. XRD pattern of iron(III) 2-ethylhexanoate pyrolysis products at 475 °C

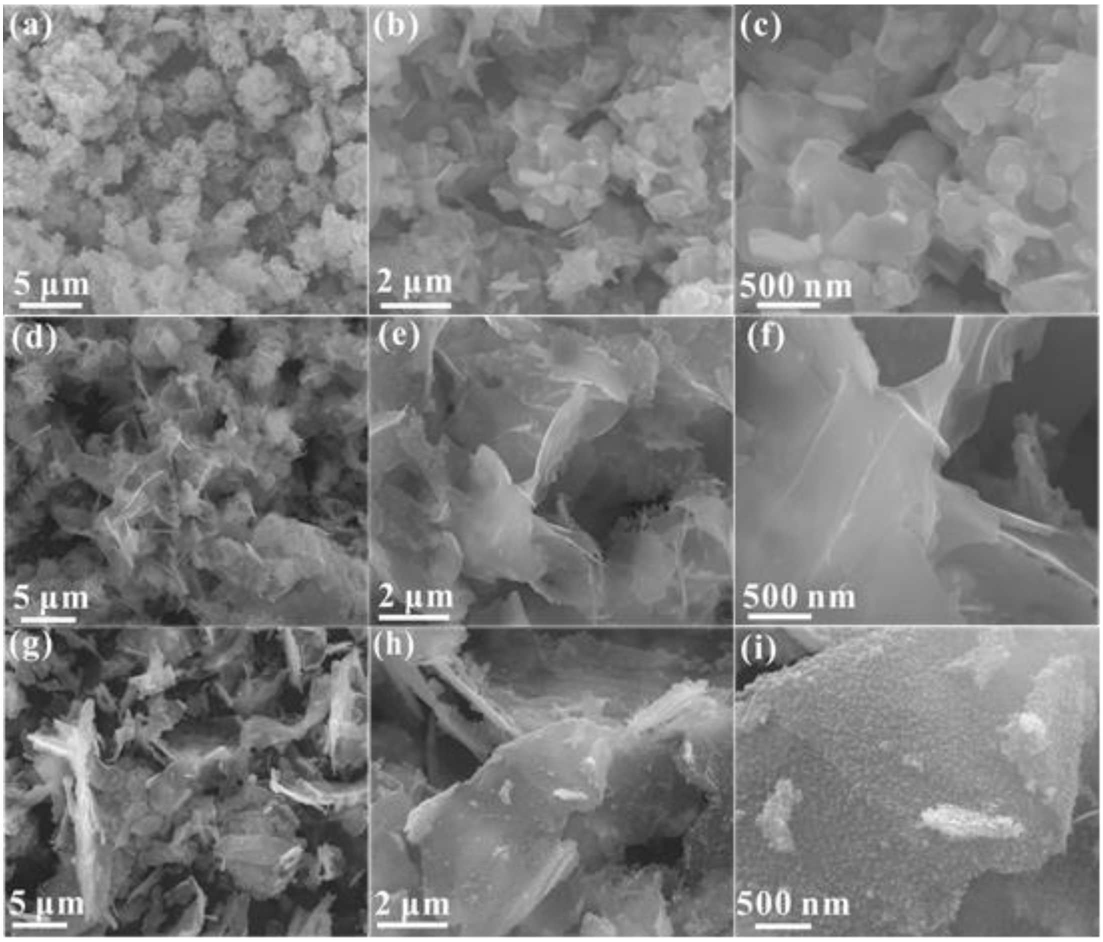

Figure S2. SEM images of Fe_3O_4/C (a, b, c), Fe_3O_4/C@G-I (d, e, f), and Fe_3O_4/C@G-II (g, h, i).

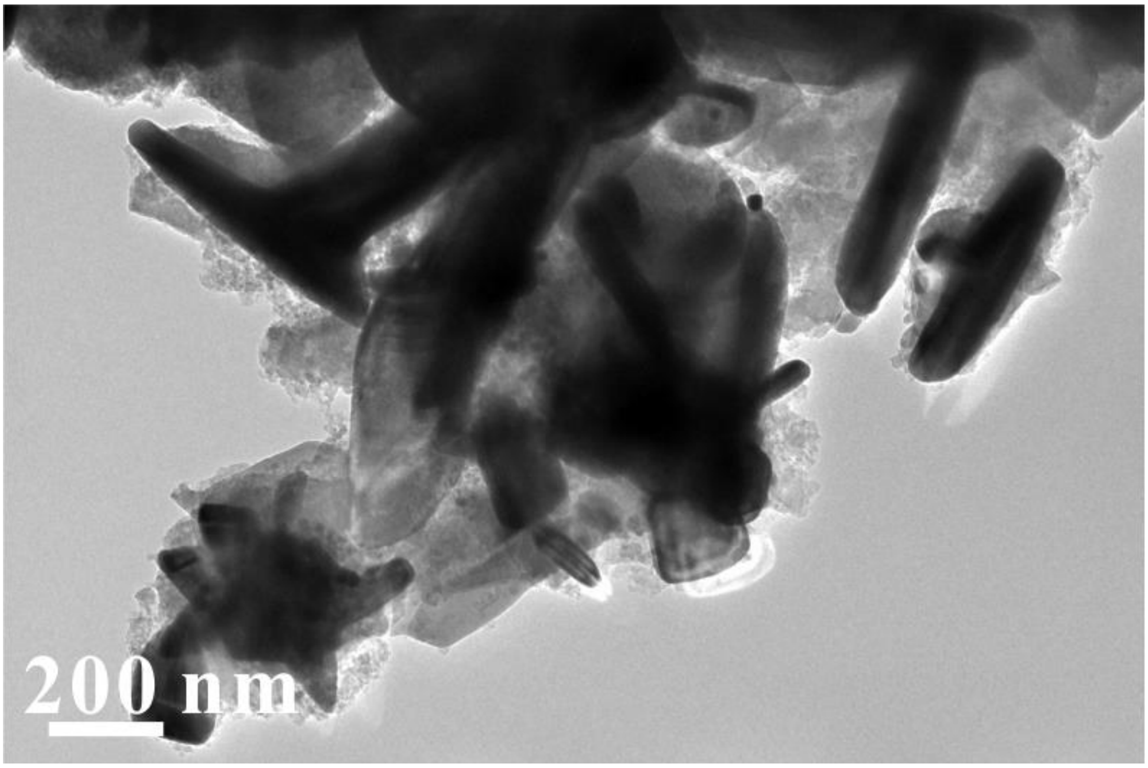

Figure S3. Low-magnification TEM image of Fe_3O_4/C composite.

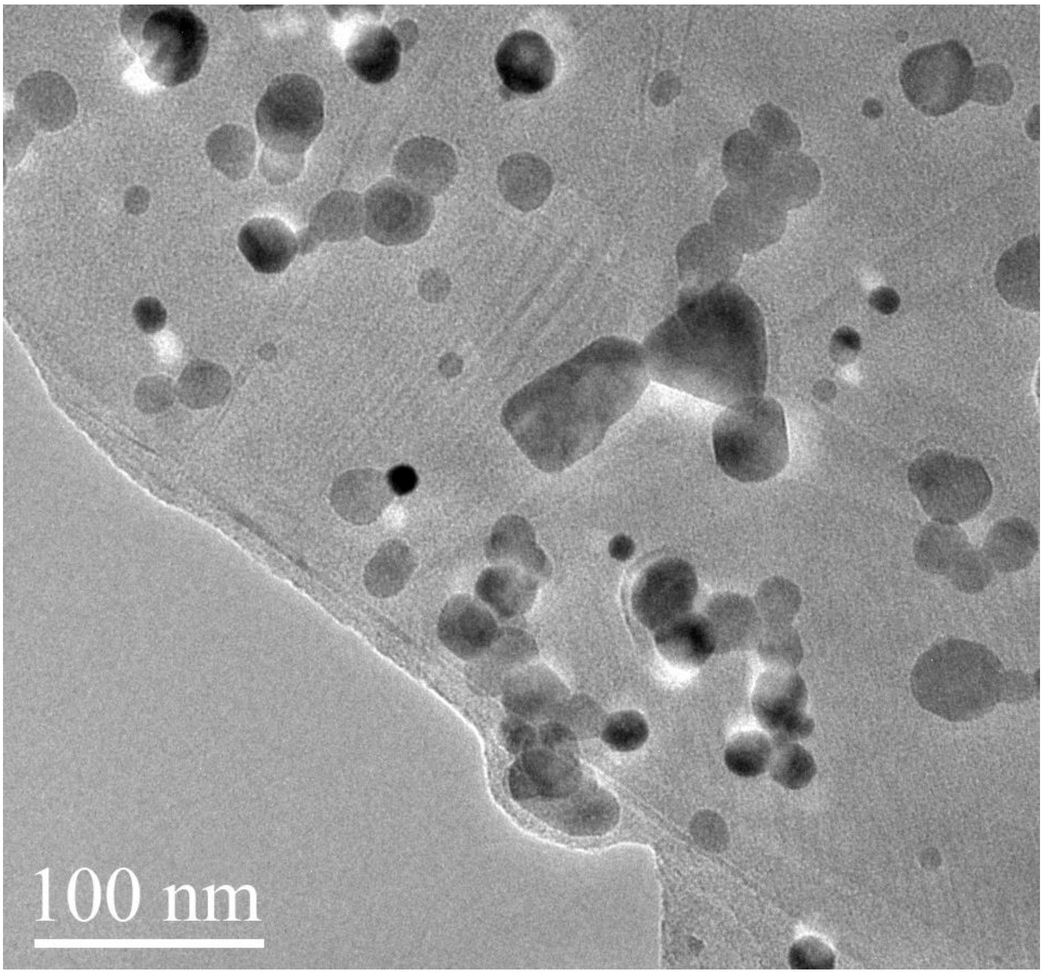

Figure S4. Low-magnification TEM image of Fe_3O_4/C@G-I composite.

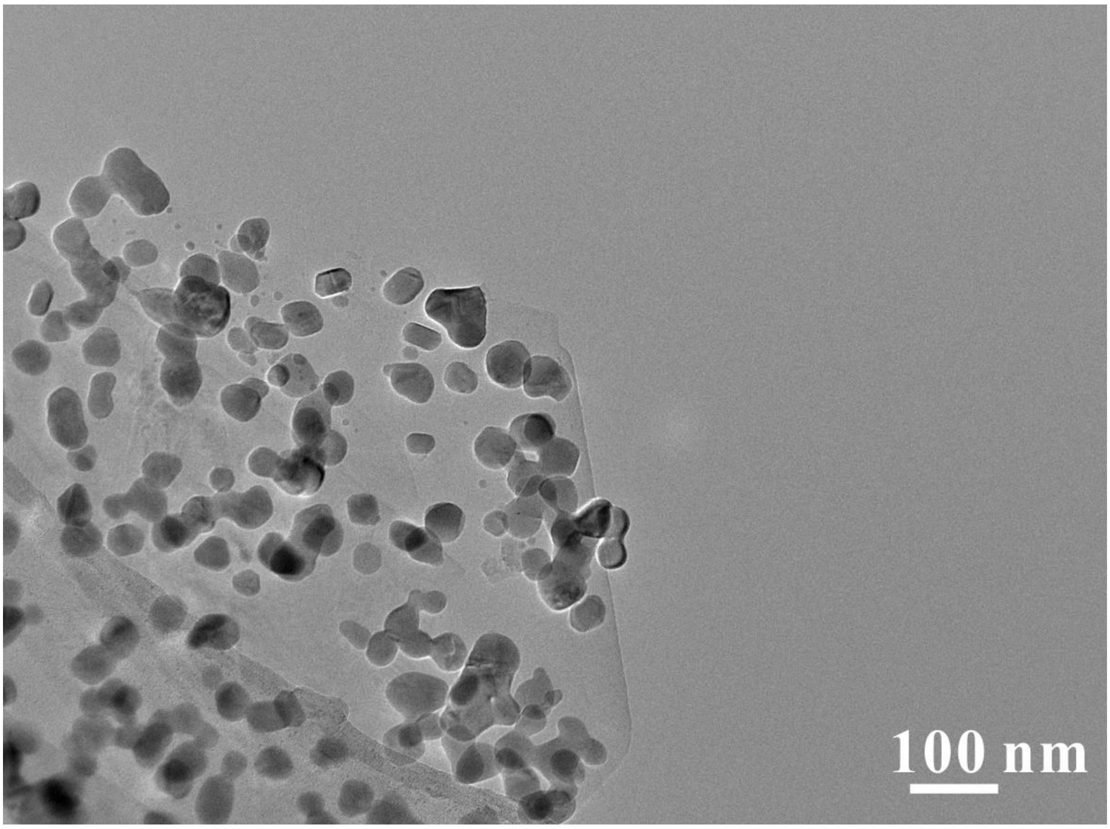

Figure S5. Low-magnification TEM image of Fe_3O_4/C@G-II composite.

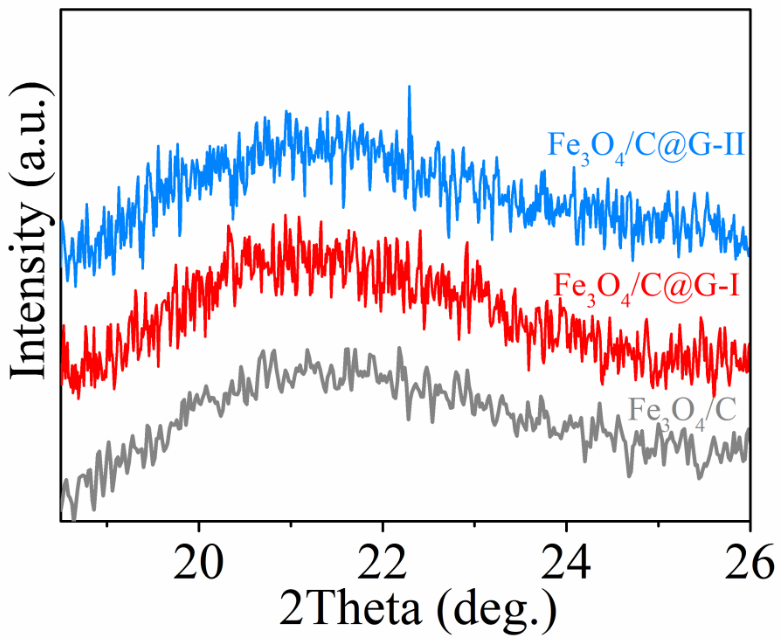

Figure S6. The localized magnification of the XRD patterns at 18.5-26° in Figure 2a.

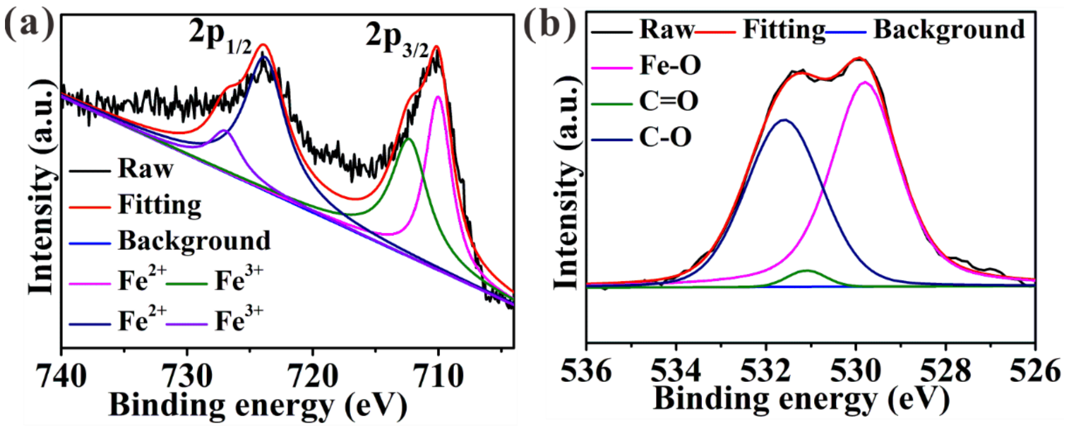

Figure S7. XPS fitting spectra of Fe (a) and O (b) in Fe_3O_4/C@G-II composite.

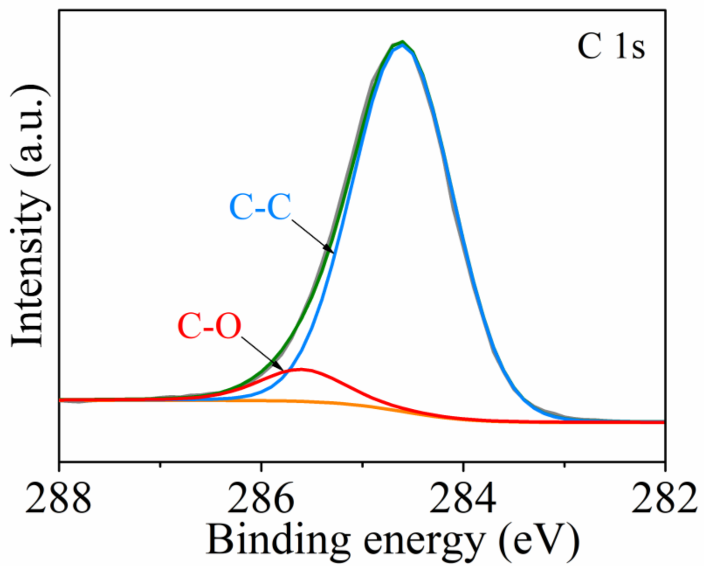

Figure S8. XPS fitting spectrum of C 1s in Fe_3O_4/C sample.

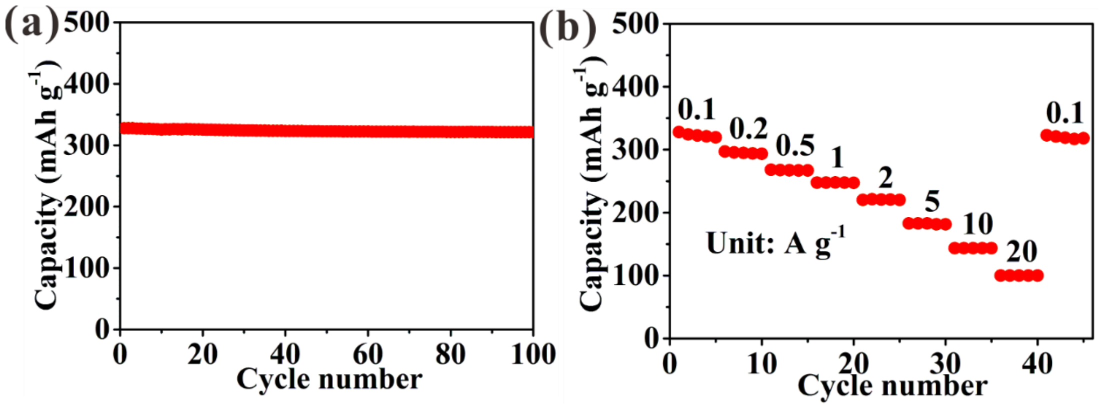

Figure S9. The cycling performance at 0.1 A g^{-1} (a) and rate capability (b) of graphene.

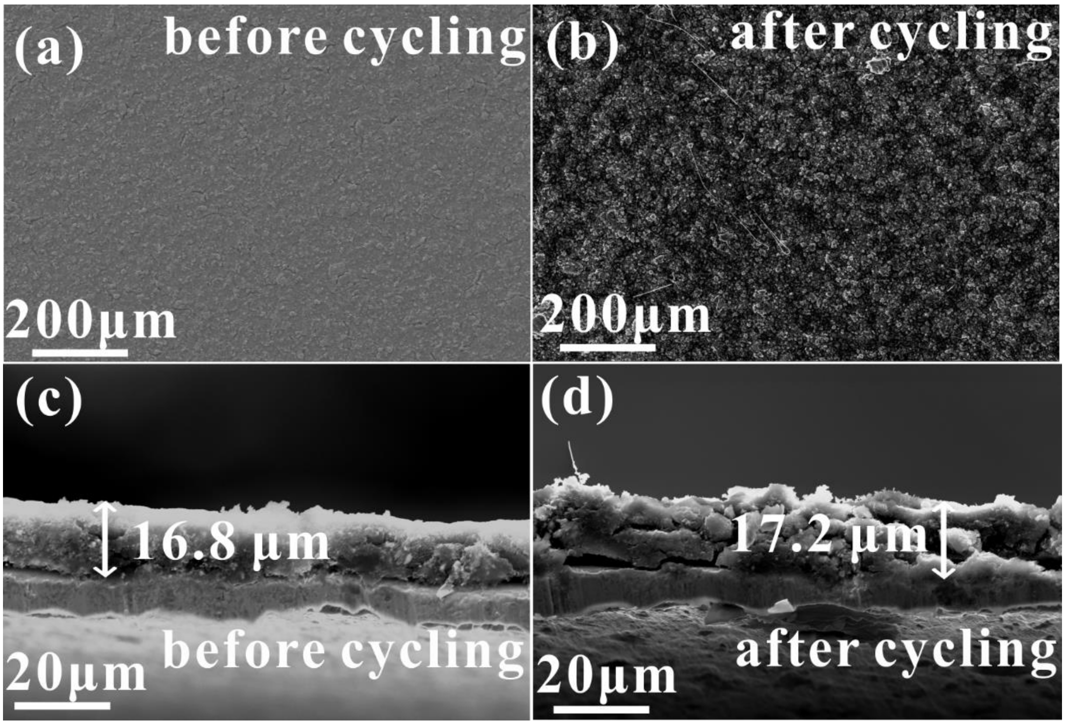

Figure S10. SEM images of Fe_3O_4/C@G-II electrodes. (a,c) before cycling and (b,d) after 100 cycling at 0.1 A g^{-1}.

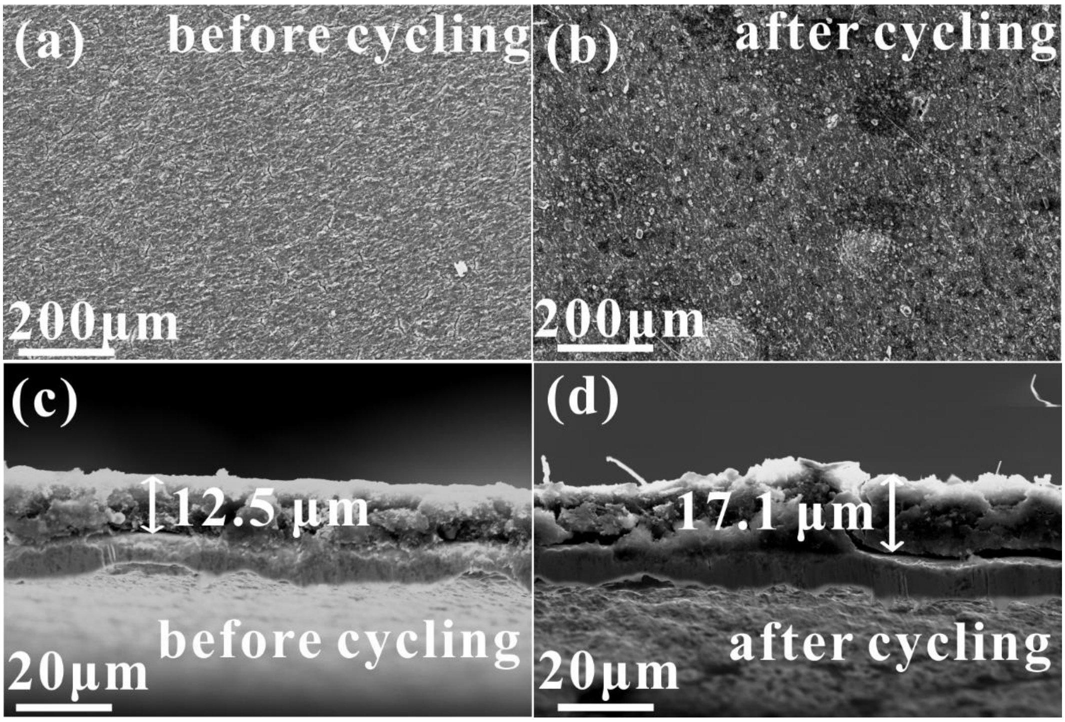

Figure S11. SEM images of Fe_3O_4/C electrodes. (a,c) before cycling and (b,d) after 100 cycling at 0.1 A g^{-1}.

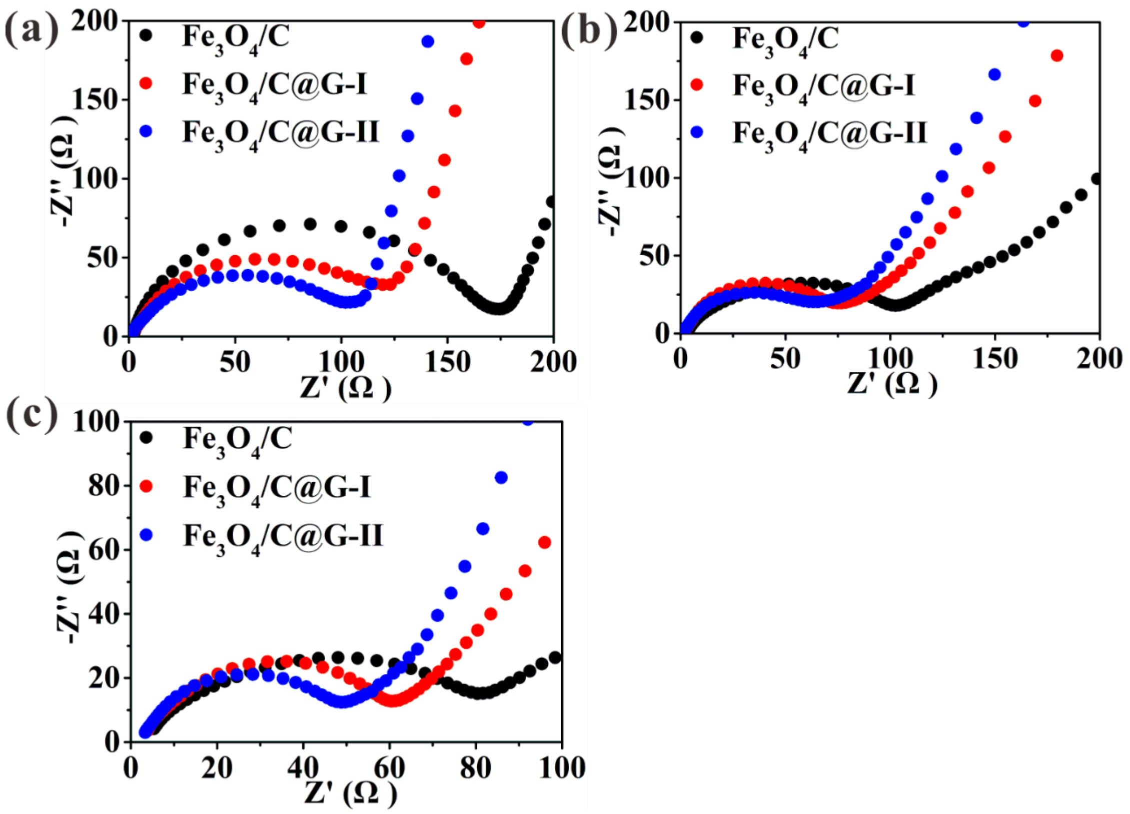

Figure S12. EIS spectra of Fe_3O_4/C, Fe_3O_4/C@G-I, and Fe_3O_4/C@G-II at different cycling number. (a) before cycling, (b) after 1st cycling, and (c) after rate tests.

To further reveal the reason for the excellent rate capability of Fe_3O_4/C@G-II, the electrochemical impedance spectroscopy (EIS) study is carried out (Figures S12). For the Nyquist plots, the diameter of the semicircle reflects the charge transfer resistance (R_{ct}), the slope of the sloping line corresponds to the Warburg impedance (W) of Na^+ diffusion, and the value of the intersection with the *x*-axis is related to the electrolyte resistance (R_s).[1] Fe_3O_4/C@G-II delivers the lowest R_{ct}, R_s, and the largest straight-line slop compared to Fe_3O_4/C and Fe_3O_4/C@G-II, indicating superior charge transport capability. These results strongly support the best rate capability of Fe_3O_4/C@G-II in Figure 3c. In addition, the R_{ct} of these samples continues to

decrease during cycling. This is mainly caused by the activation and stabilization of the electrode.[2]

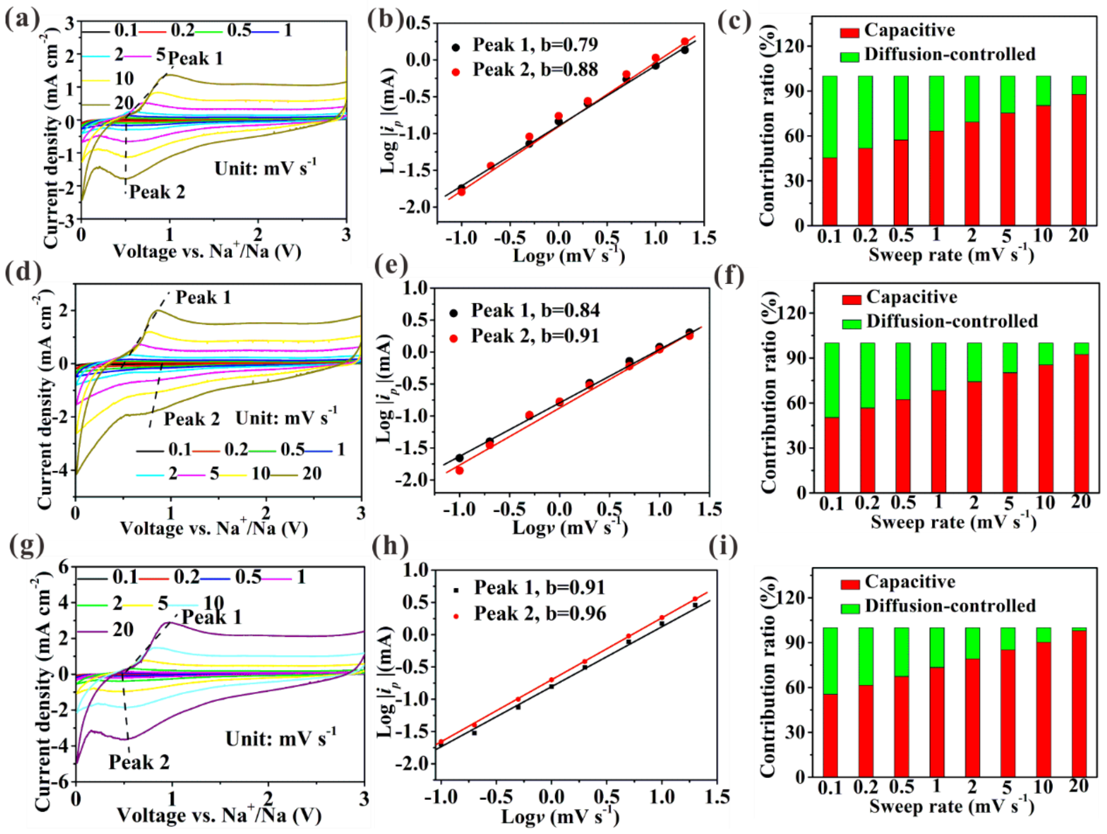

Figure S13. The electrochemical kinetic analysis of Fe_3O_4/C (a-c), Fe_3O_4/C@G-I (d-f), and Fe_3O_4/C@G-II (g-i): (a,d,g) CV curves at different sweep rates, (b,e,h) Log ip against Log v at marked peaks, (c,f,i) The percentages of pseudocapacitive contribution at different sweep rates.

The capacitive effect of the battery can be calculated based on equation (1):[3]

$$i = av^b \ldots\ldots\ldots\ldots\ldots\ldots\ldots\ldots\ldots\ldots\ldots\ldots\ldots (1)$$

where *i* and *v* represent current density and scan rate, respectively. And *a* and *b* are empirical constants, in which *b* value close to 1 represents that the system is

mainly controlled by the capacitance. For Fe_3O_4/C@G-II, b values are calculated to be 0.91 and 0.96 for peak 1 and peak 2, respectively (Figure S13h), demonstrating that the capacitive process dominates Na-ion storage dynamics. In addition, the b values of Fe_3O_4/C@G-II are larger than those of Fe_3O_4/C (0.79, peak 1; 0.88, peak 2; Figure S13b) and Fe_3O_4/C@G-I (0.84, peak 1; 0.91, peak 2; Figure S13e), suggesting that the Fe_3O_4/C@G-II electrode possesses higher capacitive contribution on capacity. The capacitive contribution can be further quantified by equation (2). [4]

$$i(V) = k_1 v + k_2 v^{1/2} \qquad (2)$$

where $i(V)$, k_1v, and $k_2v^{1/2}$ reflect total current, capacitance process, diffusion-controlled behavior, respectively. As the scan rate increases from 0.1 to 20 mV s^{-1}, the contribution of capacitance rises from 55.4% to 97.9% for the Fe_3O_4/C@G-II electrode (Figure S13c), higher than Fe_3O_4/C (45.3 to 87.8%, Figure S13c) and Fe_3O_4/C@G-I (50.2 to 92.5%, Figure S13f). The higher capacitive contribution is beneficial for obtaining higher capacity and better rate capability.

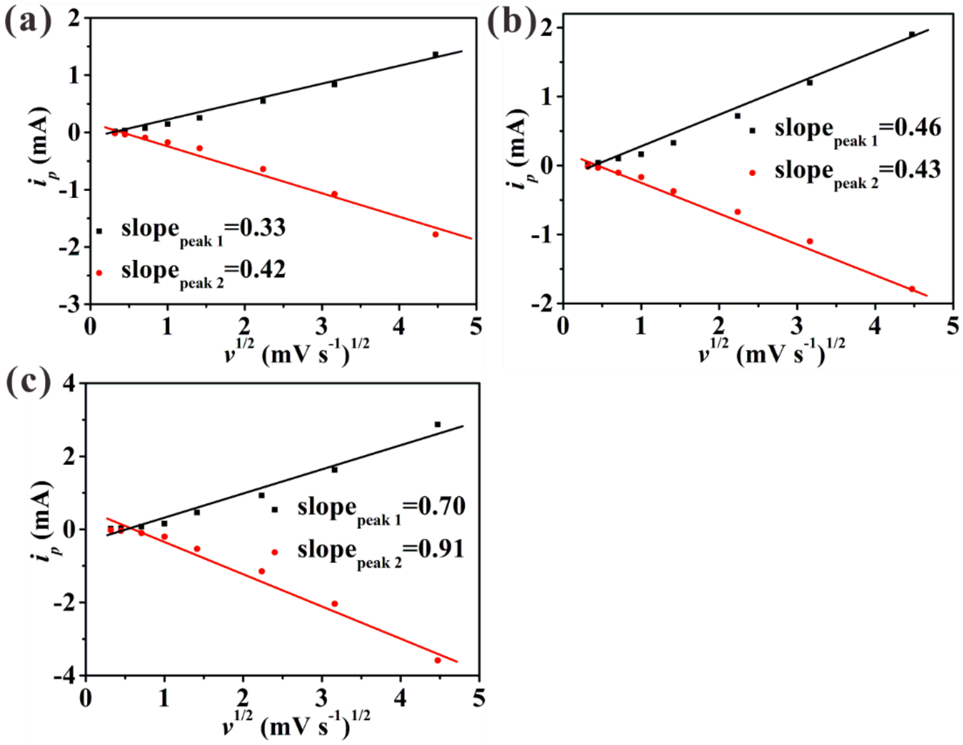

Figure S14. i_p versus $v_{1/2}$ at peaks 1 and 2 for Fe_3O_4/C composite (a), Fe_3O_4/C@G-I composite (b), and Fe_3O_4/C@G-II composite (c).

To better understand the electrochemical kinetics, the Na-ion diffusion coefficient (D_{Na^+}) could be calculated by the following equation.[5]

$$i_p = 2.69 \times 10^5 n^{3/2} A D_{Na+}^{1/2} C_{Na+} v^{1/2} \quad (3)$$

where i_p is the peak current, v is the scan rate, A is the contact area between active materials and electrolyte, n is the number of electrons involved in the reaction, and C_{Na+} is the Na^+ bulk concentration. Based on the linear relationship between i_p and $v^{1/2}$ of the redox peaks (Figure S14), the D_{Na+} values for Peaks 1 and 2 are obtained (Peak 1 – 0.24*10^{-10} cm^2 s^{-1}, Peak 2 – 0.38*10^{-10} cm^2 s^{-1}, Fe_3O_4/C; Peak 1 – 0.62*10^{-10} cm^2 s^{-1}, Peak 2 – 0.42*10^{-10} cm^2 s^{-1}, Fe_3O_4/C@G-I; Peak 1 – 0.85*10^{-10} cm^2 s^{-1}, Peak 2 – 1.53*10^{-9} cm^2 s^{-1}, Fe_3O_4/C@G-II), which indicates an ultrafast Na^+ transport and thereby the superior rate capability for Fe_3O_4/C@G-II.

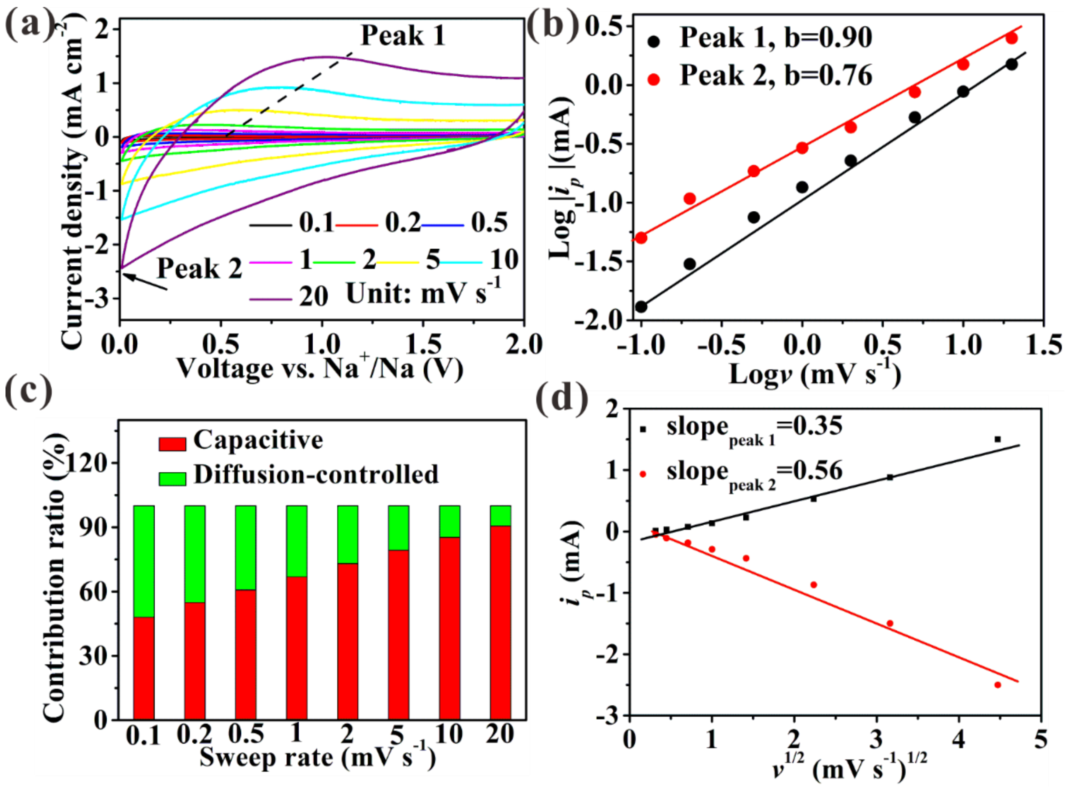

Figure S15. The electrochemical kinetic analysis of graphene: (a) CV curves at different sweep rates, (b) Log i_p against Log v at marked peaks, (c) The percentages of pseudocapacitive contribution at different sweep rates, and (d) i_p versus $v^{1/2}$ at peaks 1 and 2.

For graphene, *b* values are calculated to be 0.90 and 0.76 for peak 1 and peak 2, respectively (Figure S15b), demonstrating that the capacitive process dominates Na-ion storage dynamics. The *b* values of graphene are lower than those of Fe_3O_4/C@G-I (0.84, peak 1; 0.91, peak 2; Figure S13e) and Fe_3O_4/C@G-II (0.91, peak 1; 0.96, peak 2; Figure S13h), indicating the high capacitive contribution is mainly depend on the nanosized Fe_3O_4/C nanoparticles in Fe_3O_4/C@G-I and Fe_3O_4/C@G-II. This is also confirmed by the comparison of the capacitance contribution of graphene (rises from 46.3% to 87.9%, Figure S15c) with Fe_3O_4/C@G-I and Fe_3O_4/C@G-II as the scan rate increases from 0.1 to 20 mV s^{-1}. In

addition, the D_{Na+} values of graphene for Peaks 1 and 2 are 0.31*10^{-10} cm^2 s^{-1} and 0.73*10^{-10} cm^2 s^{-1}, respectively, further confirming this point.

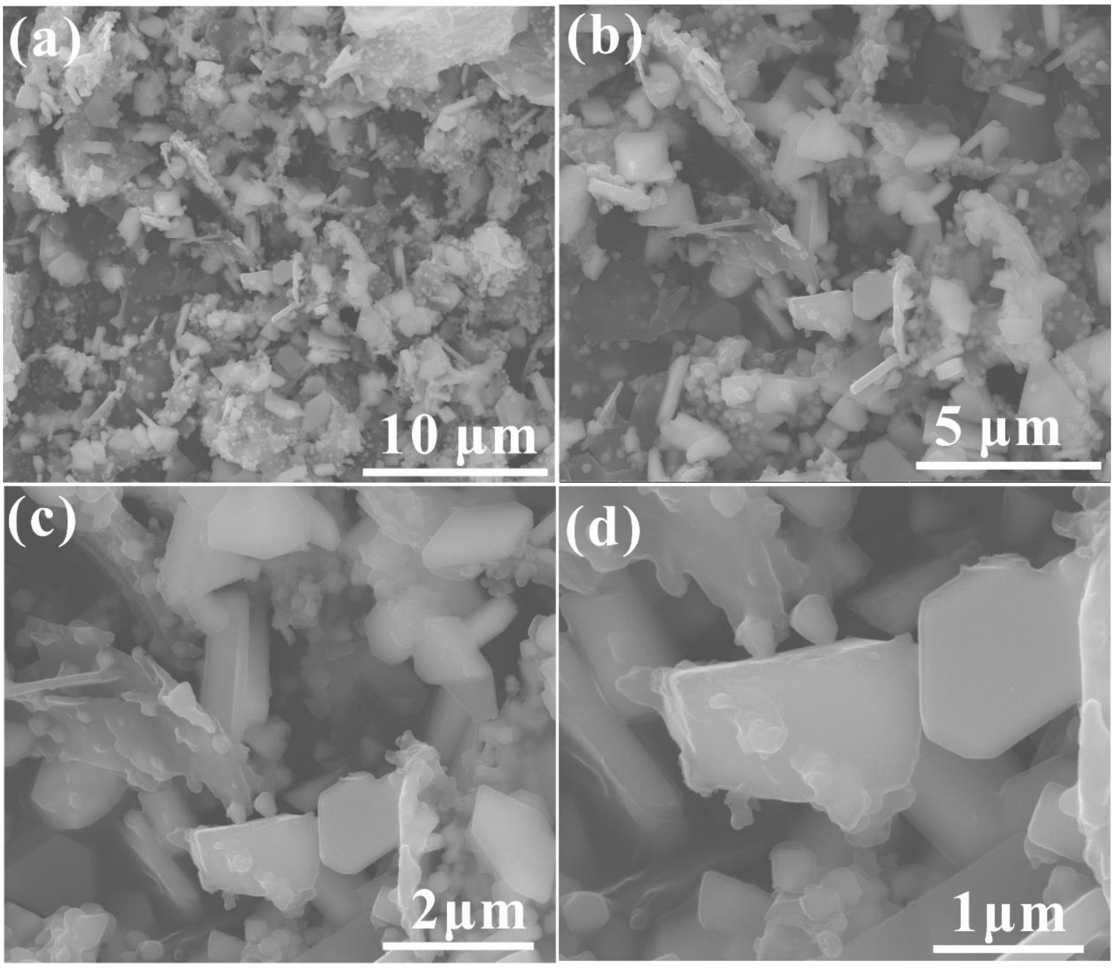

Figure S16. SEM images of Fe_3O_4/C@G-III with excessive iron isooctanoate.

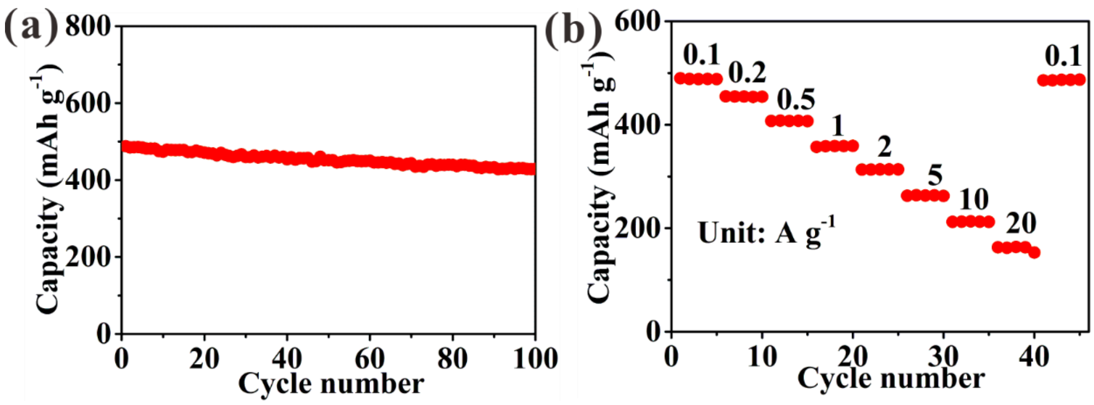

Figure S17. The cycling performance at 0.1 A g^{-1} (a) and rate capability (b) of Fe_3O_4/C@G-III with excessive iron isooctanoate.

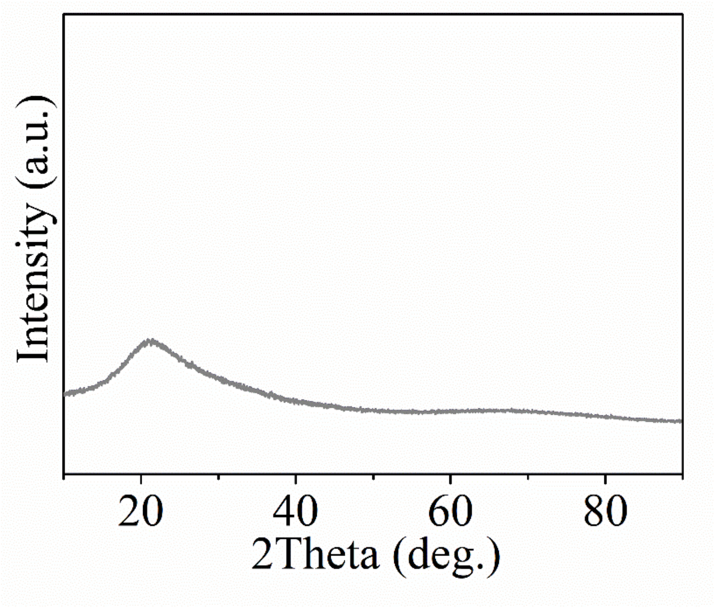

Figure S18. XRD pattern of Fe_3O_4/C@G-II after HCl etching.

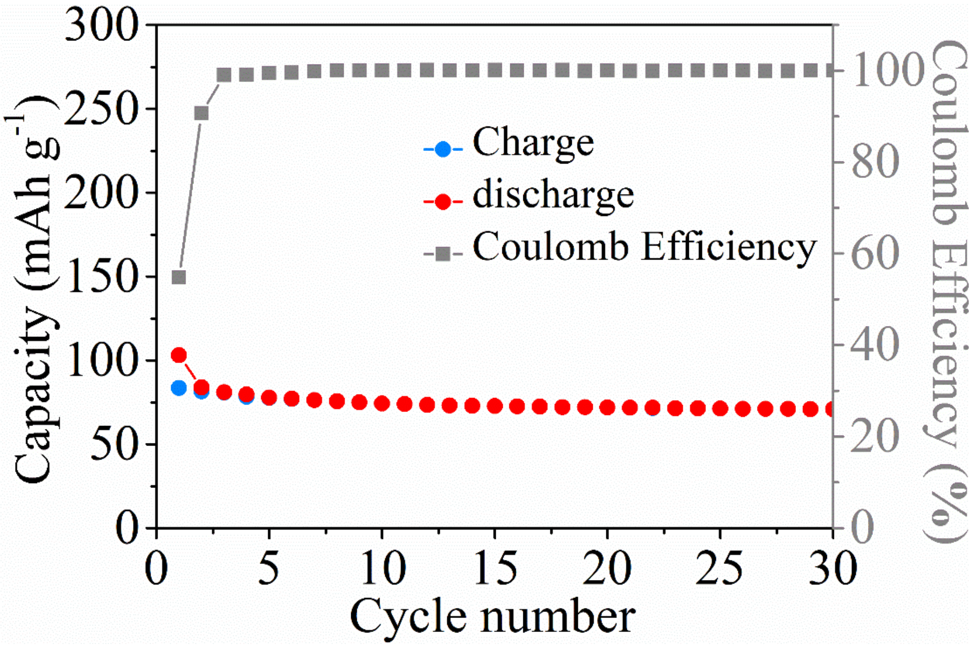

Figure S19. Cycle performance of HCl etched Fe_3O_4/C@G-II at -40 ℃.

Table S1 Comparison of electrochemical performances of Fe_3O_4/C@G-II with previously reported anode materials for SIBs **at low temperature** in open reports. C_C-first charge capacity (mAh g^{-1}), T-operating temperature (℃), C_R-capacity retention (%), M_L-mass loading (mg cm^{-2}), J-current density (A g^{-1}), N_C-cycle number, NA-not available.

Samples	C_C	T	C_R	M_L	J	N_C	Rate	References
**Fe_3O_4/C@G-II**	**276.5**	**-40**	**99.3**	**1.1**	**0.1**	**100**	**158.6 mAh g^{-1} at 2 A g^{-1}; 131.3 mAh g^{-1} at 5 A g^{-1}; 101.3 mAh g^{-1} at 10 A g^{-1}; 74.0 mAh g^{-1} at 20 A g^{-1};**	**This work**
HCP	268	-15	81	~0.6	0.5	1000	170 mAh g^{-1} at 2.0 A g^{-1}	Advanced Materials 31.40 (2019): 1903125.
3D NTP/C-F	111	-20	94.6	~1.2	0.2	500	NA	Advanced Functional Materials 31.11 (2021): 2009458.
FeS_2@G@CNF	~430	-20	~46	NA	0.2	100	30 mAh g^{-1} at 3.0 A g^{-1}	Small 15.10 (2019): 1804740.
FeS_2@G@CNF	~550	0	~45	NA	0.2	100	65 mAh g^{-1} at 3.0 A g^{-1}	Small 15.10 (2019): 1804740.

F-$CuFeS_2$@RGO	~200	-40	91	~1.1	2	210	NA	Nano Research (2023): 1-9.
Bi	~180	-40	~89	1.7	0.2	40	NA	Angewandte Chemie International Edition 61.13 (2022): e202116930.
PANI/$Ti_3C_2T_x$	~200	-30	~50 %	~1	0.1	100	NA	ACS applied materials & interfaces 12.35 (2020): 39181-39194.
a-MoO_2/MoS_2@NC	~190	-40	~80	NA	0.1	100	~20 mAh g^{-1} at 4.0 A g^{-1};	Inorganic Chemistry Frontiers 9.24 (2022): 6391-6403.
a-MoO_2/MoS_2@NC	~230	-20	~95	NA	0.1	100	~30 mAh g^{-1} at 4.0 A g^{-1};	Inorganic Chemistry Frontiers 9.24 (2022): 6391-6403.
$FeSe_2$/rGO	271	-40	53	0.8	1	200	NA	Chemical Engineering Journal 422 (2021): 130054.

P–NiSe@C	548.5	-5	57.2	0.79	0.2	50	NA	Composites Part B: Engineering 179 (2019): 107538.
NbSSe	136	0	92.67	1.5	0.03	500	85 mAh g^{-1} at 3.0A g^{-1};	Chemical Engineering Journal 435 (2022): 134838.
NTP/C–CNTs	113	-20	97	NA	0.1	100	62 mAh g^{-1} at 10C;	RSC Adv. 2016, 6, 70277
$NaTi_2(PO_4)_3$/C foams	125	-20	87	NA	0.2	500	95 mAh g^{-1} at 20C;	Advanced Functional Materials 2020, 31, 2009458.
ZnSe@NCNFs	305	-20	69.3	NA	0.2	200	90 mAh g^{-1} at 5 A g^{-1};	ACS Sustainable Chem. 2021, 9, 11705.
Ti_3C_2-N funct	190	-25	77	NA	1	2000	90 mAh g^{-1} at 5 A g^{-1};	RSC Advances 2016, 6, 70277.
Bi@3DCF	220	-20	80	NA	1	100	NA	Mater. Today Energy 2021, 20, 100627.

Graphite	70	-40	54	NA	NA	NA	59 mAh g^{-1} at 0.3 A g^{-1};	Angew. Chem., Int. Ed. 2021, 60, 23858.
MoS_2@MXene @D-TiO_2	~250	-30	~65	1	0.05	100	NA	ACS Applied Materials & Interfaces 14.14 (2022): 16300-16309.

Table S2 Comparison of electrochemical performances of Fe_3O_4/C@G-II with previously reported anode materials for SIBs **at room temperature** in open reports. C_C-first charge capacity (mAh g^{-1}), C_R-capacity retention (%), M_L-mass loading (mg

cm^{-2}), J-current density (A g^{-1}), N_C-cycle number, NA-not available.

Samples	C_C	C_R	M_L	J	N_C	References
**Fe_3O_4/C@G-II**	**516.9**	**101**	**1.1**	**0.1**	**100**	**This work**
**Fe_3O_4/C@G-II**	**406.1**	**109.6**	**1.1**	**1**	**2000**	**This work**
**Fe_3O_4/C@G-II**	**345.5**	**111.7**	**1.1**	**5**	**6000**	**This work**
NCS@NDDC	437.5	86.7	2	0.5	100	Advanced Functional Materials 28.47 (2018): 1805444.
HCP	308	93	~0.6	0.2	1000	Advanced Materials 31.40 (2019): 1903125.
HCP	~240	74	~0.6	2	1000	Advanced Materials 31.40 (2019): 1903125.
Zn-HC CE	335	77	NA	2	5000	Advanced Materials (2023): 2211461.
ACGC900	~250	~90	~1.8	0.05	200	Nano-micro letters 13 (2021): 1-14.

PANI/$Ti_3C_2T_x$	~310<br>~180	~82<br>75	~1<br>~1	0.1<br>2	100<br>10000	ACS applied materials & interfaces 12.35 (2020): 39181-39194.
PANI/$Ti_3C_2T_x$	~180	75	~1	2	10000	ACS applied materials & interfaces 12.35 (2020): 39181-39194.
FeS_2@G@CNF	~440	91.2	NA	0.1	100	Small 15.10 (2019): 1804740.
FeS_2@G@CNF	~328	93	NA	3	2400	Small 15.10 (2019): 1804740.
a-MoO_2/MoS_2@NC	432.5	94	NA	0.1	100	Inorganic Chemistry Frontiers 9.24 (2022): 6391-6403.
a-MoO_2/MoS_2@NC	~300	94.2	NA	1	1500	Inorganic Chemistry Frontiers 9.24 (2022): 6391-6403.
$Ni_{1.5}CoSe_5$@NC@rGO	573.2	91	~1.1	0.5	100	Applied Surface Science 620

						(2023): 156836.
$Ni_{1.5}CoSe_5$@NC@rGO	~490	~92	~1.1	5	220	Applied Surface Science 620 (2023): 156836.
F-$CuFeS_2$@RGO	450	96	~1.1	5	4800	Nano Research (2023): 1-9.

Table S3 Comparison of electrochemical performances of Fe_3O_4/C@G-II with previously reported anode materials for SIBs **at high temperature** in open reports. C_C-first charge capacity (mAh g^{-1}), T-operating temperature temperature (℃), C_R-capacity retention (%), M_L-mass loading (mg cm^{-2}), J-current density (A g^{-1}), N_C-cycle number, NA-not available.

Samples	C_C	T	C_R	M_L	J	N_C	References
**Fe_3O_4/C@G-II**	**540.7**	**60**	**98.1**	**1.1**	**0.1**	**100**	**This work**
PANI/$Ti_3C_2T_x$	~400	50	~80	~1	0.1	100	ACS applied materials & interfaces 12.35 (2020): 39181-39194.
Bi	~320	60	~81	1.7	4	100	Angewandte Chemie International Edition 61.13 (2022): e202116930.
P–NiSe@C	893.3	55	63.8	0.79	0.2	50	Composites Part B: Engineering 179 (2019): 107538.

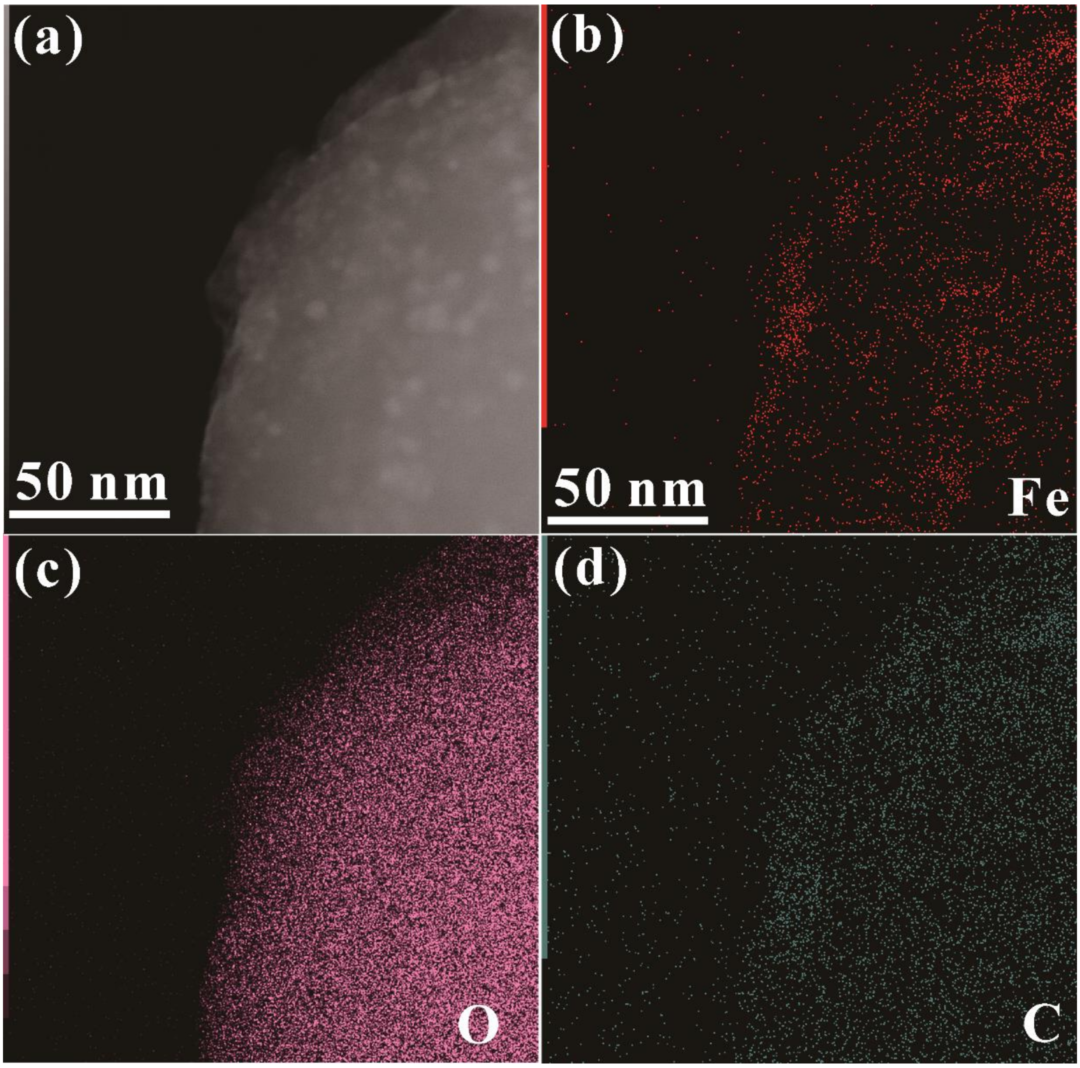

Figure S20. HAADF image (a) and the corresponding EDS elemental mapping images of Fe_3O_4/C@G-II electrode after discharging to 0.01 V at -40°C.

.

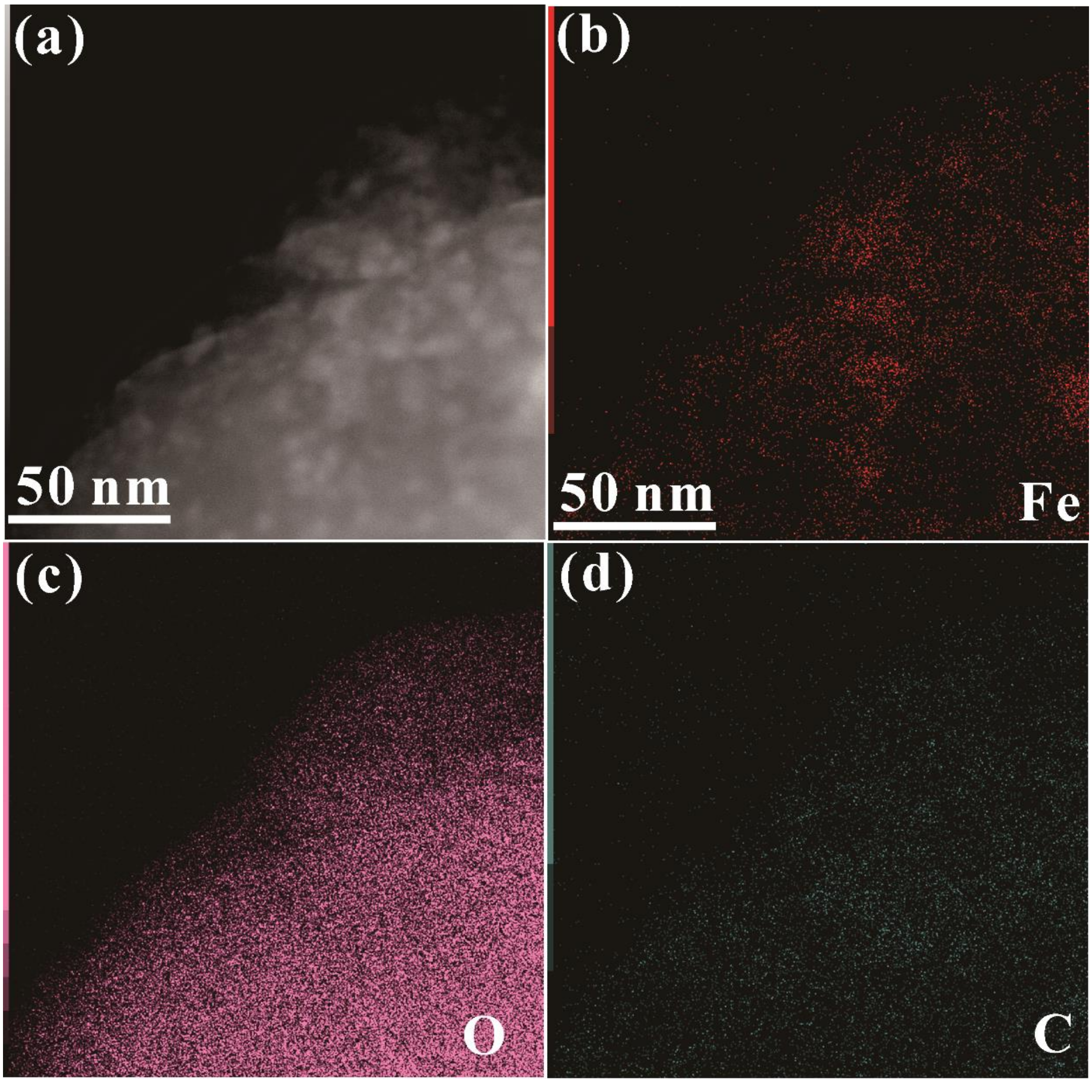

Figure S21. HAADF image (a) and the corresponding EDS elemental mapping images of Fe_3O_4/C@G-II electrode after discharging to 0.01 V at 25°C.

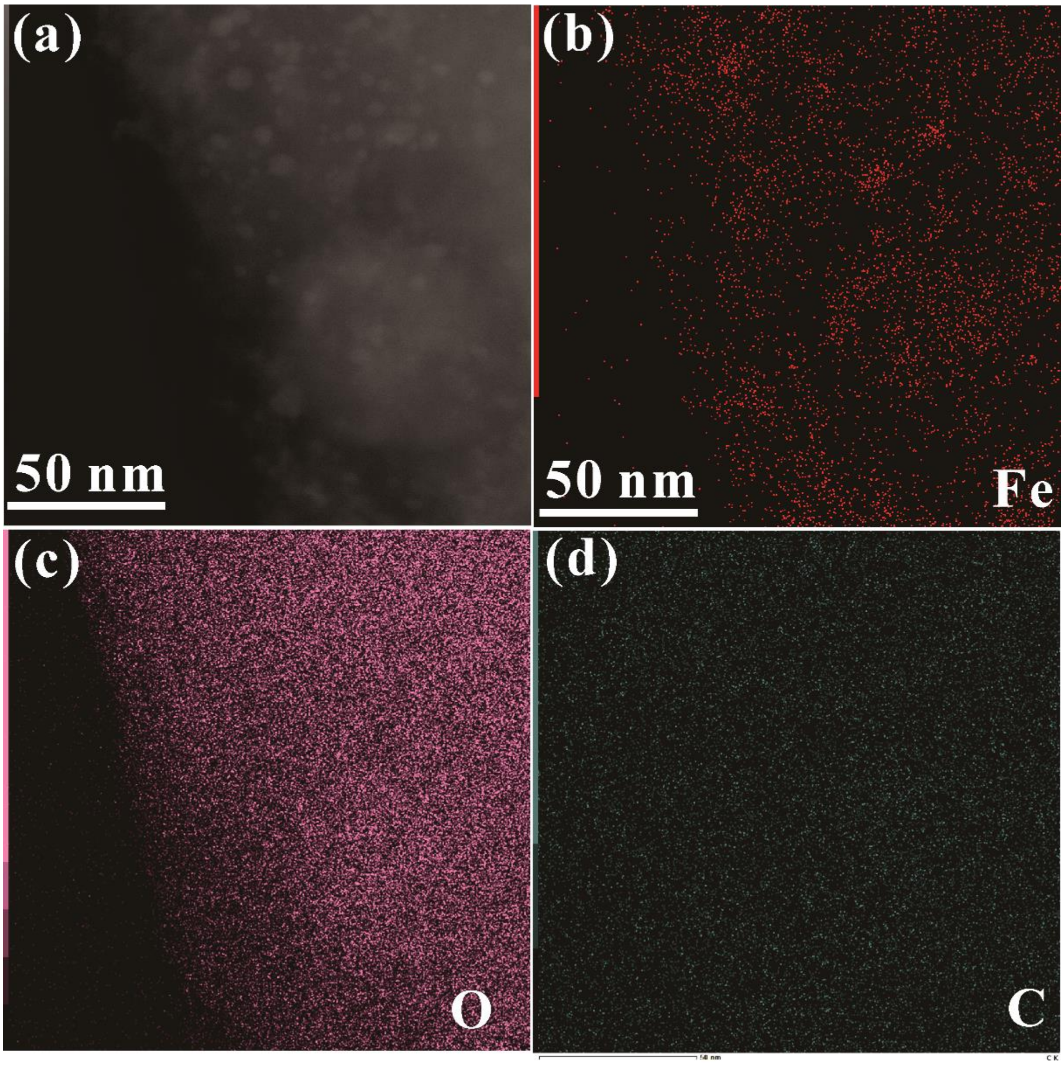

Figure S22. HAADF image (a) and the corresponding EDS elemental mapping images of Fe_3O_4/C@G-II electrode after discharging to 0.01 V at 60°C.

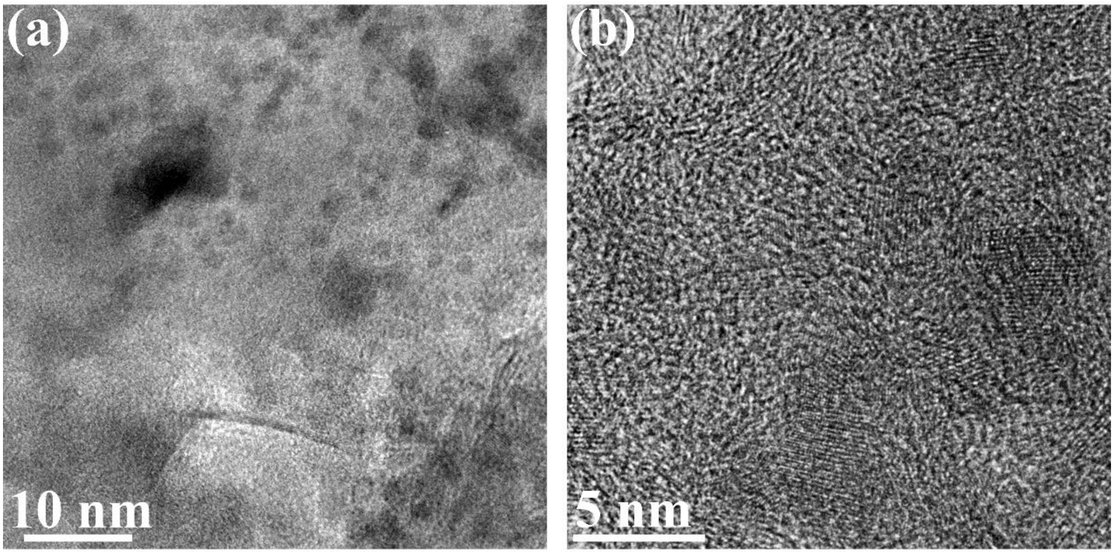

Figure S23. (a) TEM and (b) HRTEM images of Fe_3O_4/C@G-II electrode after recharging to 3 V at -40 °C.

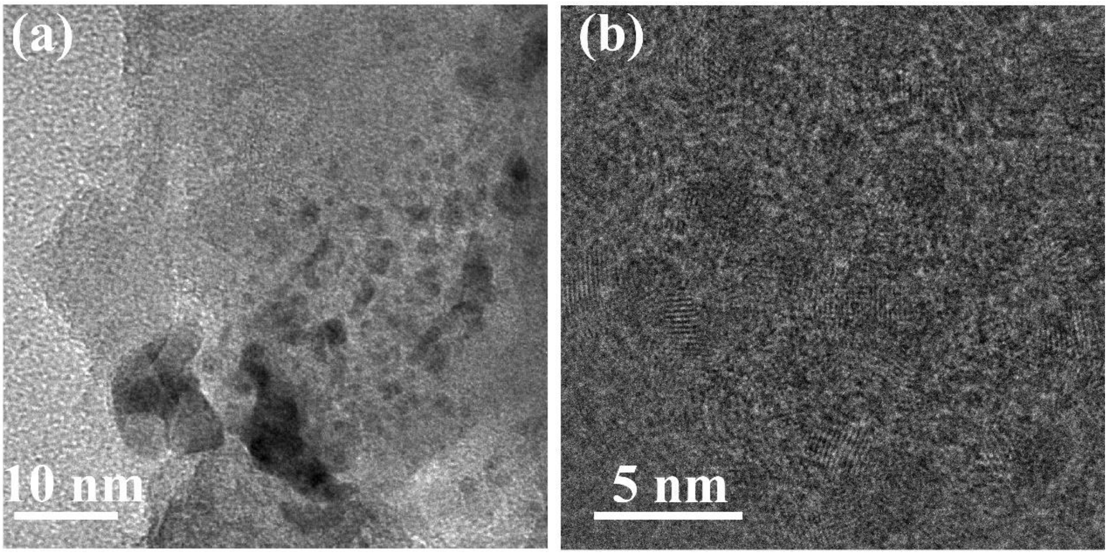

Figure S24. (a) TEM and (b) HRTEM images of Fe_3O_4/C@G-II electrode after next discharging to 0.01 V at -40 °C.

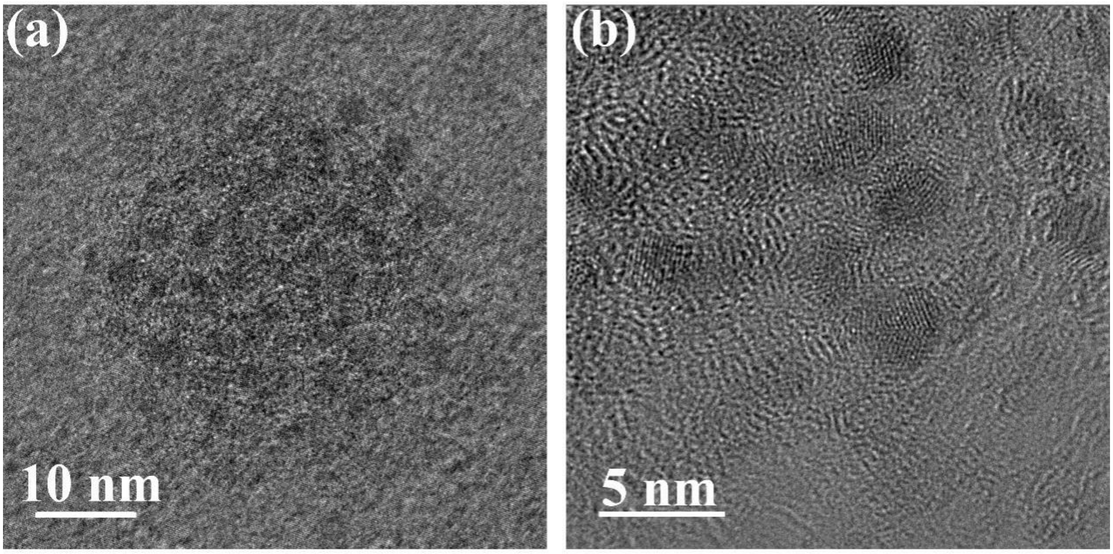

Figure S25. (a) TEM and (b) HRTEM images of Fe_3O_4/C@G-II electrode after recharging to 3 V at 25 °C.

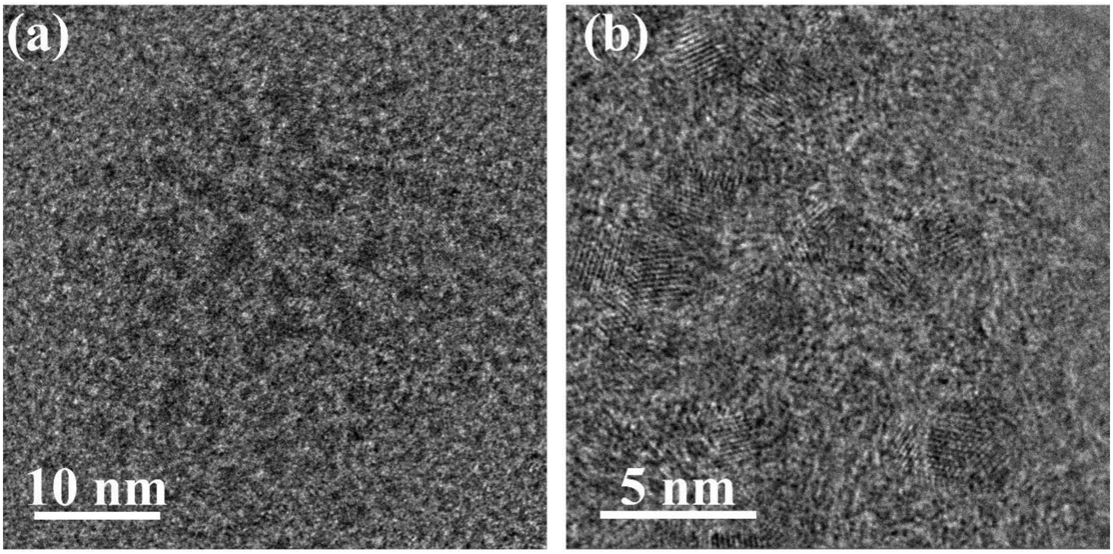

Figure S26. (a) TEM and (b) HRTEM images of Fe_3O_4/C@G-II electrode after next discharging to 0.01 V at 25 °C.

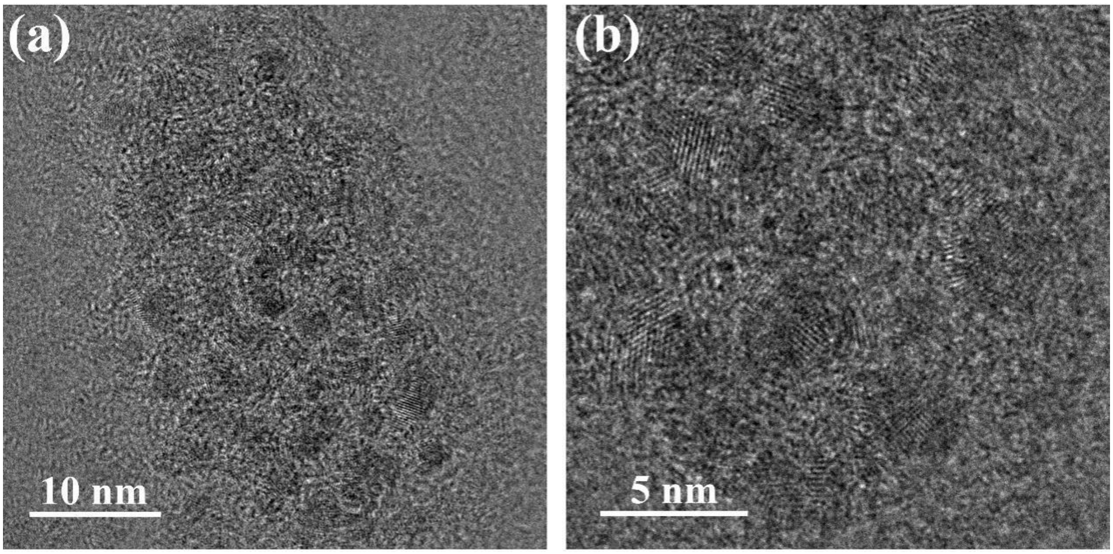

Figure S27. (a) TEM and (b) HRTEM images of Fe_3O_4/C@G-II electrode after recharging to 3 V at 60 °C.

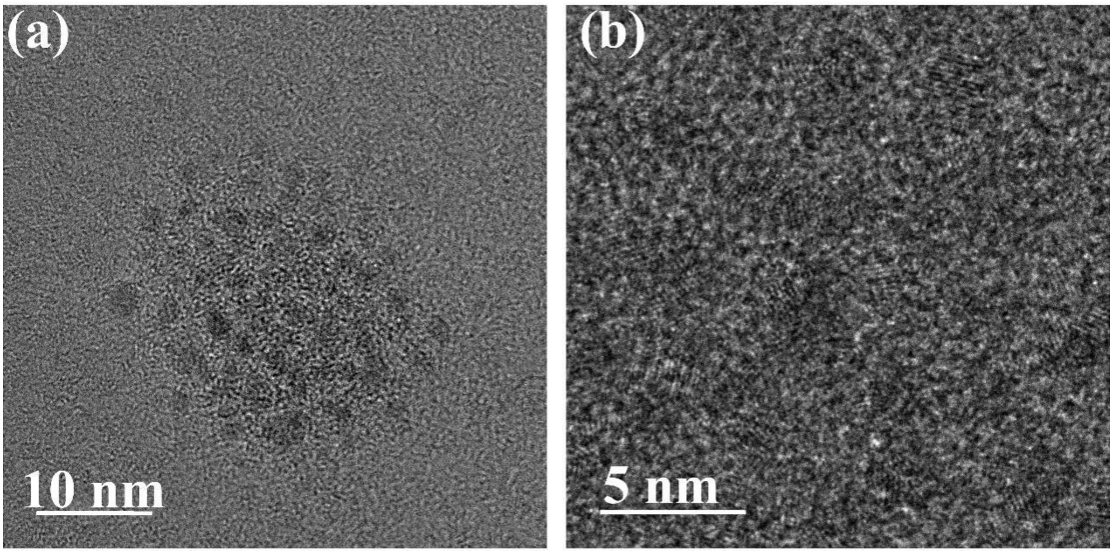

Figure S28. (a) TEM and (b) HRTEM images of Fe_3O_4/C@G-II electrode after next discharging to 0.01 V at 60 °C.

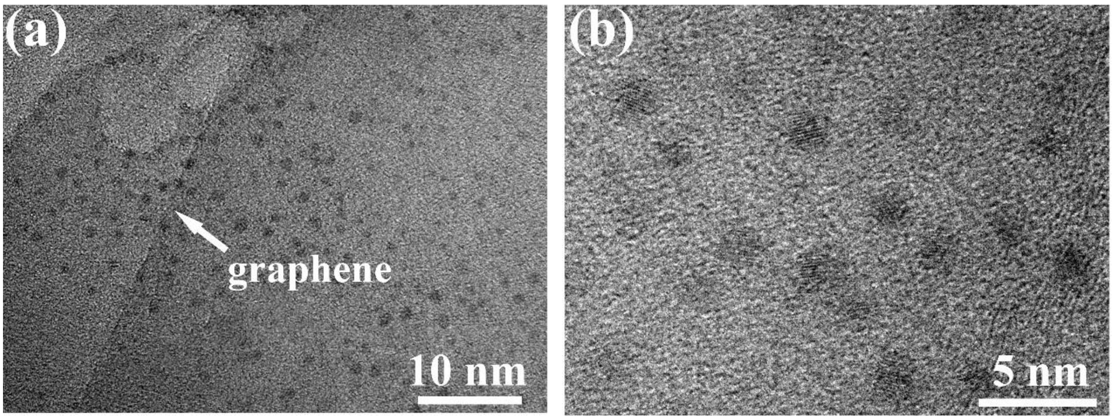

Figure S29. (a) TEM and (b) HRTEM images of Fe_3O_4/C@G-II electrode after 100 cycles at 0.1 A g^{-1} at -40 °C.

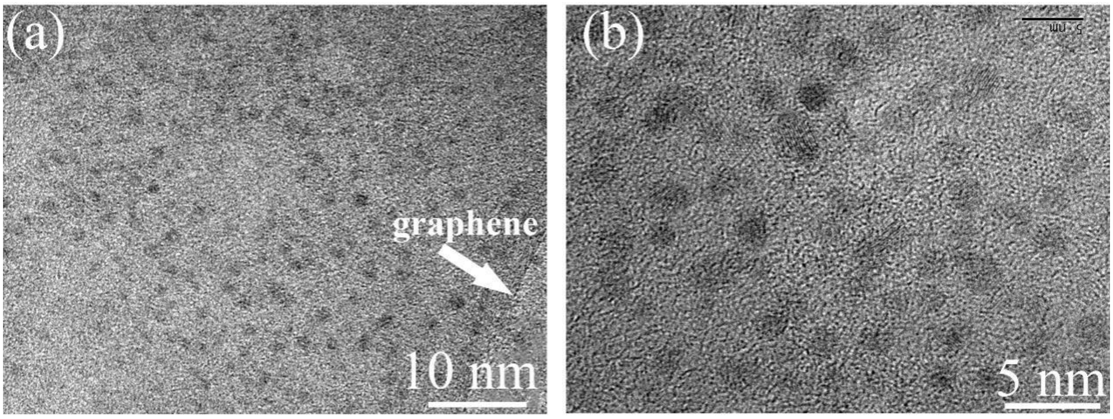

Figure S30. (a) TEM and (b) HRTEM images of Fe_3O_4/C@G-II electrode after 100 cycles at 0.1 A g^{-1} at 25 °C.

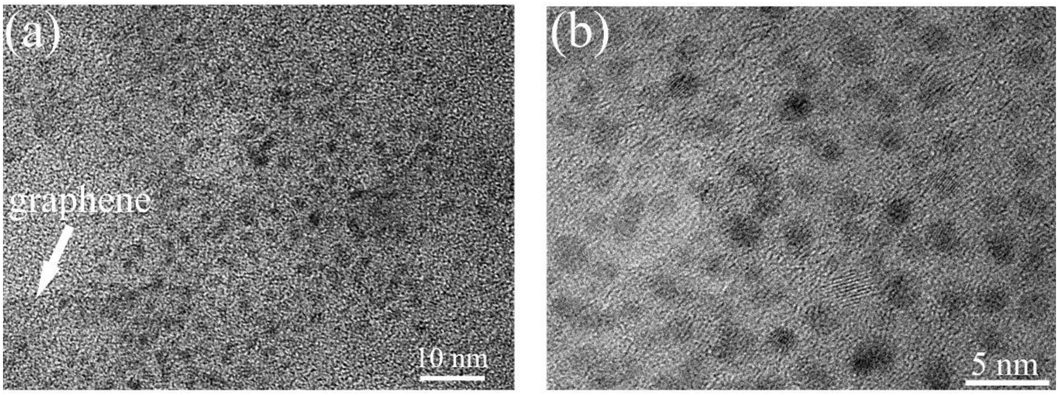

Figure S31. (a) TEM and (b) HRTEM images of Fe_3O_4/C@G-II electrode after 100 cycles at 0.1 A g^{-1} at 60 °C.

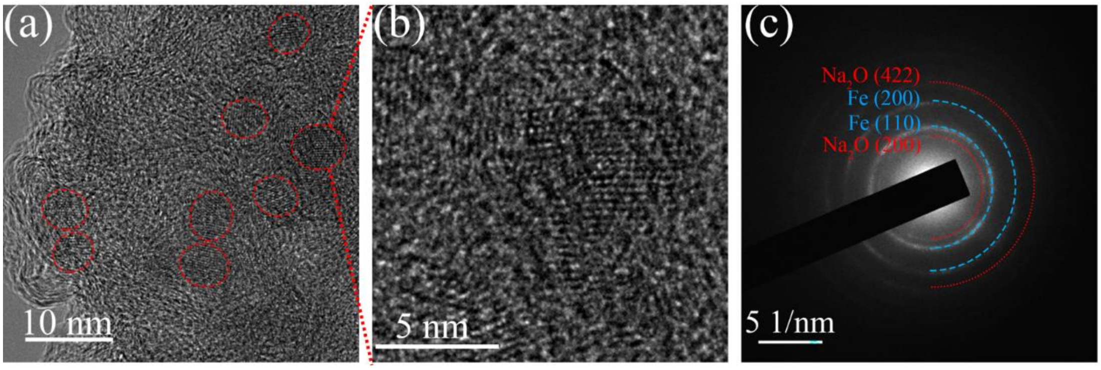

Figure S32. (a, b) HRTEM images of Fe_3O_4/C electrode after discharging to 0.01 V at 25°C. (c) SAED patterns of Fe_3O_4/C@ electrode after discharging to 0.01 V at 25 °C.

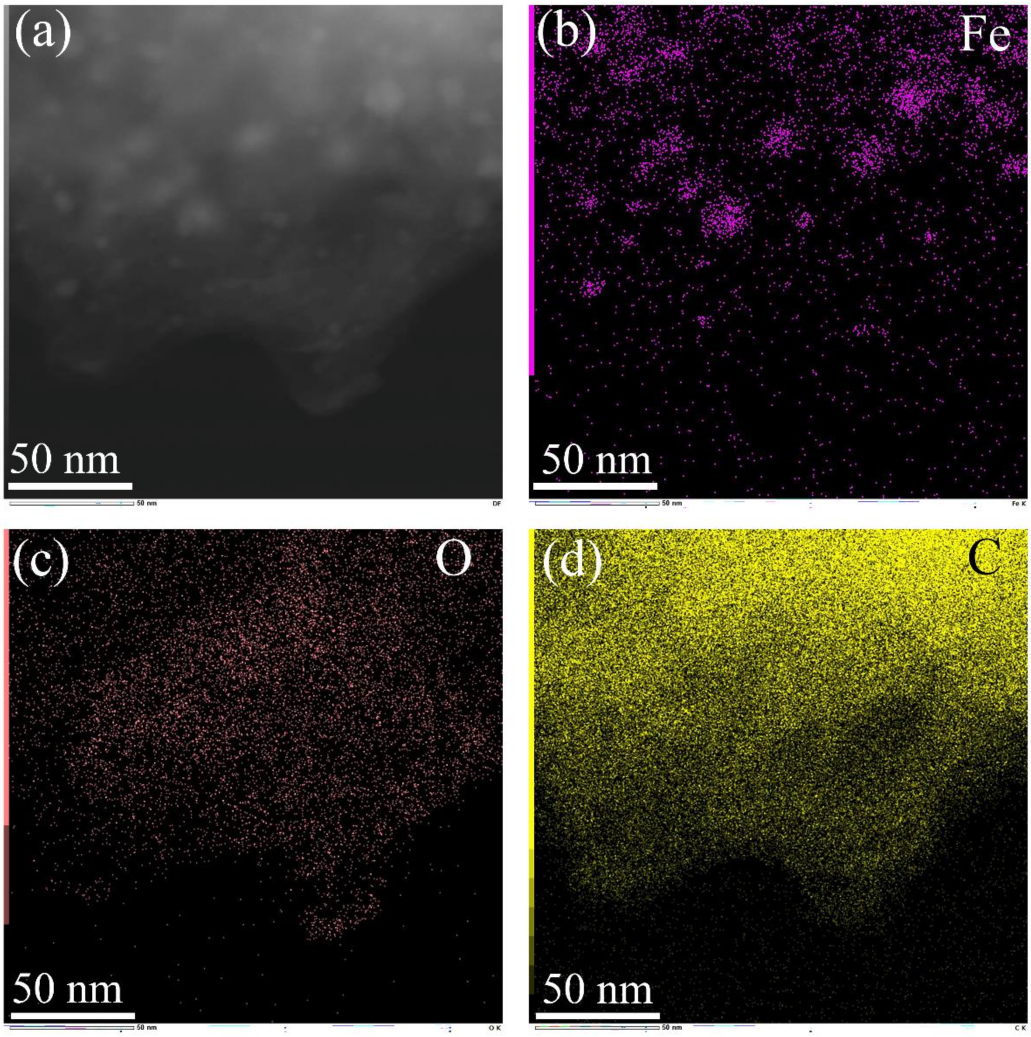

Figure S33. HAADF image (a) and the corresponding EDS elemental mapping images of Fe_3O_4/C electrode after discharging to 0.01 V at 25°C.

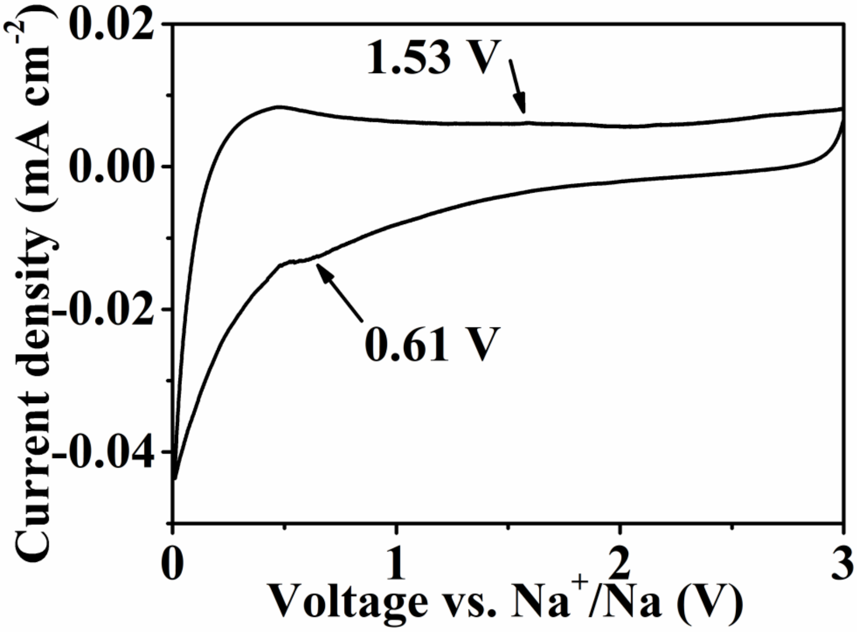

Figure S34. CV curves of Fe_3O_4/C@G-II electrode with scan rate of 0.1 mV s^{-1} at -40°C.

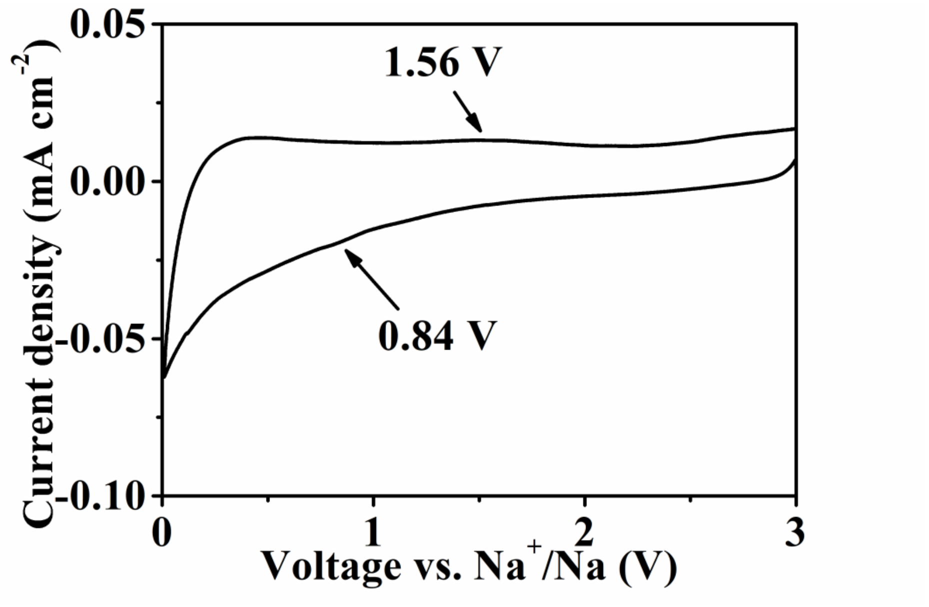

Figure S35. CV curves of Fe_3O_4/C@G-II electrode with scan rate of 0.1 mV s^{-1} at 25°C.

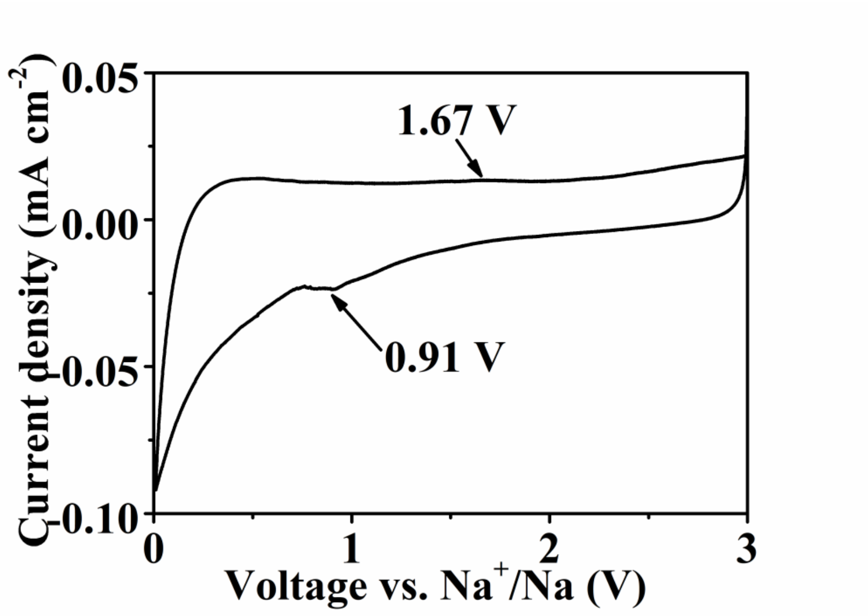

Figure S36. CV curves of Fe_3O_4/C@G-II electrode with scan rate of 0.1 mV s^{-1} at 60°C.

Table S4 Comparison of electrochemical performances of Fe_3O_4/C@G-II with previously reported full cell in SIBs **at room temperature** in open reports. C_C-first charge capacity (mAh g^{-1}), T-operating temperature temperature (°C), C_R-capacity retention (%), M_L-mass loading (mg cm^{-2}), J-current density (C), N_C-cycle number, NA-not available. Note that the large difference in C_C depends mainly on whether the capacity is calculated based on anode or cathode.

Samples	C_C	T	C_R	M_L	J	N_C	References
**Fe_3O_4/C@G-II**	**111.3**	**25**	**98.6**	**1.1**	**0.1**	**100**	**This work**
**Fe_3O_4/C@G-II**	**93.7**	**25**	**75.7**	**1.1**	**1**	**500**	**This work**
NCS@NDDC	374.5	25	97.6	2	0.4	60	Advanced Functional Materials 28.47 (2018): 1805444.
PANI/$Ti_3C_2T_x$	~ 230	25	65	~1	0.1	100	ACS applied materials & interfaces 12.35 (2020): 39181-39194.
FeS_2@G@CNF	105	25	~87	NA	1	100	Small 15.10 (2019): 1804740.
Zn-HC CE		25		NA	0.1		Advanced Materials (2023): 2211461.
$Ni_{1.5}CoSe_5$@NC @rGO	~275	25	~89	~1.1	1	70	Applied Surface Science 620 (2023): 156836.
$Ni_{1.5}CoSe_5$@NC @rGO	~200	25	~59	~1.1	1	70	Applied Surface Science 620 (2023): 156836.
Fe_3O_4@CNT	~110 0	25	~40	1.5	0.1	200	Journal of Materials Chemistry A 4.47 (2016): 18532-18542.
Fe_3O_4@NCm/r GO	~340	25	~56	NA	0.1	50	Journal of Alloys and Compounds 832 (2020): 154879.

References

1 J. Ruan, F. Mo, Z. Chen, M. Liu, S. Zheng, R. Wu, F. Fang, Y. Song, D. Sun, *Adv. Energy Mater.*, 2020, **10**, 1904045.

2 G. Fang, Z. Wu, J. Zhou, C. Zhu, X. Cao, T. Lin, Y. Chen, C. Wang, A. Pan, S. Liang, *Adv. Energy Mater.*, 2018, **8**, 201703155.

3 G. Zhao, Y. Zhang, L. Yang, Y. Jiang, Y. Zhang, W. Hong, Y. Tian, H. Zhao, J. Hu, L. Zhou, H. Hou, X. Ji, L. Mai, *Adv. Funct. Mater.*, 2018, **28**, 201803690.

4 S.-H. Qi, J.-W. Deng, W.-C. Zhang, Y.-Z. Feng, J.-M. Ma, *Rare Metals*, 2020, **39**, 970-988.
